# Characterization of human mesenchymal stem cell secretome at early steps of adipocyte and osteoblast differentiation

**DOI:** 10.1186/1471-2199-9-26

**Published:** 2008-02-26

**Authors:** Chiara Chiellini, Olivia Cochet, Luc Negroni, Michel Samson, Marjorie Poggi, Gérard Ailhaud, Marie-Christine Alessi, Christian Dani, Ez-Zoubir Amri

**Affiliations:** 1 ISBDC, Université de Nice Sophia-Antipolis, CNRS ; 28 avenue de Valrose, 06100 Nice, France; 2 IFR 50, Faculté de Médecine, Plate-Forme Protéomique, Avenue de Valombrose, 06107 Nice, Cedex 02, France; 3 INSERM, Unité 638, Faculté de Médecine, Université de Nice Sophia Antipolis, Avenue de Valombrose, 06107 Nice, Cedex 02, France; 4 INSERM UMR 626; Faculté de Médecine Timone, 27 Boulevard Jean Moulin, 13385 Marseille, Cedex 5, France

## Abstract

**Background:**

It is well established that adipose tissue plays a key role in energy storage and release but is also a secretory organ and a source of stem cells. Among different lineages, stem cells are able to differentiate into adipocytes and osteoblasts. As secreted proteins could regulate the balance between both lineages, we aimed at characterizing the secretome of human multipotent adipose-derived stem cell (hMADS) at an early step of commitment to adipocytes and osteoblasts.

**Results:**

A proteomic approach, using mono-dimensional electrophoresis and tandem mass spectrometry, allowed us to identify a total of 73 proteins at day 0 and day 3 of adipocyte and osteoblast differentiation. Analysis of identified proteins showed that 52 % corresponded to classical secreted proteins characterized by a signal peptide, that 37 % previously described in the extracellular compartment were devoid of signal peptide and that 11 % neither exhibited a signal peptide nor had been previously described extracellularly. These proteins were classified into 8 clusters according to their function. Quantitative analysis has been performed for 8 candidates: PAI-1, PEDF, BIGH3, PTX3, SPARC, ENO1, GRP78 and MMP2. Among them, PAI-1 was detected at day 0 and day 3 of osteoblast differentiation but never in adipocyte secretome. Furthermore we showed that PAI-1 mRNA was down-regulated in the bone of ovariectomized mice.

**Conclusion:**

Given its regulation during the early events of hMADS cell differentiation and its status in ovariectomized mice, PAI-1 could play a role in the adipocyte/osteoblast balance and thus in bone diseases such as osteoporosis.

## Background

Adipose tissue is no longer considered as a mere energy reservoir but it plays also an endocrine role, releasing a panoply of secreted molecules, *i.e*. adipokines such as leptin, adiponectin, plasminogen activator inhibitor 1 (PAI-1), vaspin and tumor necrosis factor α (TNFα) [1,2]. Furthermore, adipose tissue is a source of stem cells, representing a promising tool for pharmacological studies and clinical applications [3]. A balanced development of adipose tissue is of crucial importance to ensure some of the most important physiological functions, including reproduction, haemostasis, angiogenesis, blood pressure and immune function [1,4]. Alterations of fat cell number and size are present in lipodystrophy and obesity that are associated to type 2 diabetes [5]. Another condition altering fat cell formation is osteoporosis, where an imbalance between adipocytes and osteoblasts in bone marrow is observed. Aging, menopause, glucocorticoid treatment or alcohol abuse can lead to an increase in bone marrow adiposity [6,7]. To date, several issues are still pending, for instance whether infiltration of fat in bone marrow causes low bone mass or is due to bone loss [6,7]. Since adipocytes and osteoblasts share the same mesenchymal precursor, the study of the adipocyte/osteoblast balance represents a worthy challenge to treat adipose tissue and bone disorders. It is well described that secreted leptin and adiponectin can affect bone formation both directly on osteoblastogenesis and indirectly by acting on osteoclastogenesis [6,8-10]. Several molecules secreted by osteoblasts such as Wnt and bone morphogenetic protein favor osteogenesis at the expense of adipogenesis [7,11], thus pointing out a crosstalk between both lineages.

During the last two decades, a large number of molecular regulators of adipogenesis and osteogenesis have been described. Among them, peroxisome proliferator-activated receptor γ (PPARγ) and CCAAT/enhancer-binding proteins (C/EBPs) are well recognized factors that play major roles in adipogenesis [12], whereas runt-related transcription factor 2 (runx2), distal-less homeobox 5 (dlx5), muscle segment homeobox 2 (msx2) and osterix represent master regulators of osteogenesis [6,13].

In the present work, we aimed at identifying molecules secreted at early step of differentiation of human mesenchymal stem cells towards adipocytes and osteoblasts. To address this point, we used a cellular model recently established in our laboratory, termed hMADS cells (human multipotent adipose tissue-derived stem cells). hMADS cells, isolated from the adipose tissue of young donors, present extensive capacities of self-renewal, clonogenicity and multipotency, as they fully differentiate into adipocytes, osteoblasts, myoblasts and chondrocytes while exhibiting a normal karyotype [14-17].

Recently, proteomic approaches have been applied to study rodent and human adipose tissue secretome using cellular models that focused mainly on late events of adipogenesis [18-22]. Moreover, with respect to early events of osteogenesis, a characterization of secreted molecules from osteoblasts has not been so far reported.

Herein we have identified 73 proteins by a proteomic approach and confirmed these findings by Western-blot for 8 candidates. These proteins and their involved pathways, in particular the plasminogen system, could play an important role in regulating the adipocyte/osteoblast balance.

## Results

### Characterization of the secretome of hMADS cells committed towards adipocytes and osteoblasts

**Figure 1:**
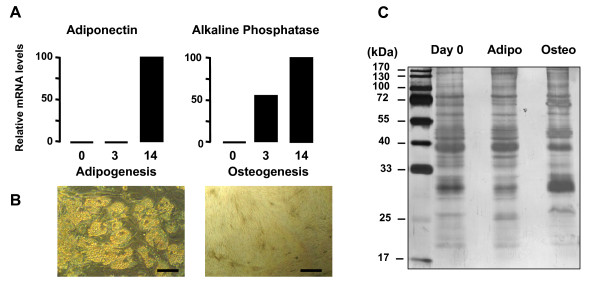
**Analysis of terminal differentiation of hMADS cells into adipocytes and osteoblasts and Coomassie Blue staining of hMADS cell secretome**. **A**. qRT-PCR analysis of mRNA levels of specific adipogenic (adiponectin) and osteogenic (alkaline phosphatase) markers at day 3 and day 14 of differentiation as compared to day 0. The data are representative of three independent experiments. **B. **Microphotographs of hMADS cells differentiated into adipocytes and osteoblasts at day 14. Bar scale = 50 μm. **C. **Representative gel of secreted proteins from hMADS cells at day 0 and day 3 of differentiating adipocytes (adipo) and osteoblasts (osteo) after 6 h of incubation. The gel is representative of 3 independent experiments.

hMADS cells differentiate into adipocytes and osteoblasts, as shown in Figure 1A. Gene expression of representative markers of adipocyte (adiponectin) and osteoblast differentiation (alkaline phosphatase) are reported and in agreement with previously published data [14-17]. The terminal differentiation of hMADS cells into adipocytes and osteoblasts is also illustrated by the typical cellular morphology as shown in Figure 1B.

In order to analyze the secretome at early step of adipogenesis and osteogenesis of hMADS cells, secretion media at day 3 of differentiation into adipocytes and osteoblasts were compared with those of cells at day 0. Cells were washed 3 times with PBS and incubated for 6 h with serum-free culture medium, containing 0.1 μg/ml of transferrin without differentiation inducers. At the end of this incubation period, media were harvested, filtered and concentrated. Very low cell death or cell lysis occurred during the incubation period, as checked by microscopic analysis and lactate dehydrogenase activity measurements [see Additional file 1]. Four μg of each protein sample were loaded onto one-dimensional SDS-PAGE and stained with Bio-Safe Coomassie Stain. A representative gel is reported in Figure 1C. The excised bands from the SDS-PAGE were cut, trypsin digested and analyzed by mass spectrometry. The list of the identified proteins is reported in Table 1. Further details about the peptides identified for each protein are available in Table 1S [see Additional file 2].

**Table 1:** Identification of proteins in the secretome of hMADS cells at early step of adipogenesis and osteogenesis.

**ID**	**Protein name**	**Function**	**Mw kDa**	**Loc**	**Day 0**	**Adipo**	**Osteo**	**Ref**
	**PROTEASES**							
P00736	Complement component 1, r subcomponent	serine protease	80	ex		Y	Y	[69]
P09871	Complement component 1, s subcomponent	serine protease	77	sp		Y	Y	
P03956	Matrix metallopeptidase 1 (interstitial collagenase)	collagen catabolism	54	sp	Y			
P08253	Matrix metallopeptidase 2, gelatinase A (MMP2)	collagen catabolism	74	sp	Y	Y	Y	
Q15113	Procollagen C-endopeptidase enhancer	collagen catabolism	48	sp		Y		
	**PROTEASE INHIBITORS**							
P05121	Serpin peptidase inhibitor, clade E, plasminogen activator inhibitor type 1(PAI-1)	fibrinolysis	45	sp	Y		Y	
P36955	Serpin peptidase inhibitor, clade F (PEDF)	inhibitor of angiogenesis	46	sp		Y		
P05155	Serpin peptidase inhibitor, clade G (C1 inhibitor), member 1	fibrinolysis	55	sp	Y	Y		
P01033	TIMP metallopeptidase inhibitor 1	proteolysis inhibitor	23	sp	Y	Y	Y	
	**EXTRACELLULAR MATRIX COMPONENTS**							
P50454	Serpin H1 precursor (Collagen-binding protein) (Colligin) (47 kDa heat shock protein) (Proliferation-inducing gene 14 protein)	collagen-binding protein	46	sp	Y			
P98160	Basement membrane-specific heparan sulfate proteoglycan core protein precursor	ECM component	469	sp	Y		Y	
P02452	Collagen, type I, alpha 1	ECM component	139	sp	Y	Y	Y	
P08123	Collagen, type I, alpha 2	ECM component	129	sp	Y	Y	Y	
P02461	Collagen, type III, alpha 1	ECM component	138	sp		Y	Y	
P12109	Collagen, type VI, alpha 1	ECM component	108	sp	Y	Y	Y	
P12110	Collagen, type VI, alpha 2	ECM component	109	sp	Y	Y	Y	
P12111	Collagen, type VI, alpha 3	ECM component	343	sp	Y	Y	Y	
P07585	Decorin precursor (Bone proteoglycan II)	ECM component	40	sp		Y		
P02751	Fibronectin precursor	cell growth	26	sp	Y	Y	Y	
Q08380	Galectin-3-binding protein precursor (Lectin galactoside-binding soluble 3-binding protein)	growth regulation/cell-matrix	65	sp	Y	Y		
Q16270	Insulin-like growth factor-binding protein 7 precursor (IGFBP-7)	cell adhesion	29	sp			Y	
P11047	Laminin gamma 1 chain precursor (Laminin B2 chain)	ECM component	178	sp	Y	Y		
P51884	Lumican precursor (Keratin sulfate proteoglycan lumican)	ECM component	38	sp	Y	Y	Y	
P14543	Nidogen 1	ECM component	136	sp		Y		
Q15063	Periostin, osteoblast specific factor	cell adhesion	93	sp	Y		Y	
Q02809	Procollagen-lysine, 2-oxoglutarate 5-dioxygenase 1 pecursor	collagen process	83	sp		Y		
O00391	Sulfhydryl oxidase 1 precursor, Quiescin Q6 (QSCN6)	growth regulation	83	sp			Y	
P09486	Secreted protein, acidic, cysteine-rich (osteonectin)	cell-matrix interaction	35	sp	Y	Y	Y	
Q15582	Transforming growth factor-beta-induced protein ig-h3 precursor (BIGH3)	cell adhesion	75	sp	Y	Y	Y	
	**ANTI-INFLAMMATORY/ANTI-OXIDANT PROTEINS**							
Q12841	Follistatin-like 1	immunity and defense	35	sp	Y	Y	Y	
P09211	Glutathione S-transferase P	antioxidant	23	ex		Y		[70, 71]
P26022	Pentraxin-related gene, rapidly induced by IL-1 beta (PTX3)	inflammatory response	42	sp	Y	Y	Y	
P30041	PRDX6 peroxiredoxin 6	antioxidant	25	ex		Y		[72]
Q16881	TXNRD1 thioredoxin reductase 1	antioxidant	55	ex		Y		[33]
	**METABOLIC ENZYMES**							
Q04828*	Aldo-keto reductase family 1, member C1 (dihydrodiol dehydrogenase 1)	progesterone conversion	37	in		Y		
P06733	Enolase 1 (ENO1)	glycolysis	47	ex	Y	Y	Y	[73, 74]
P04406	Glyceraldehyde-3-phosphate dehydrogenase	glycolysis	36	ex	Y	Y	Y	[31]
P40926	Malate dehydrogenase 2, NAD (mitochondrial)	Krebs cycle/gluconeogenesis	36	ex		Y	Y	[75, 76]
P00558	Phosphoglycerate kinase 1	glycolysis	44	ex		Y		[32]
P18669	Phosphoglycerate mutase 1	glycolysis	29	ex		Y		[70]
P14618	Pyruvate kinase, muscle	glycolysis	58	ex	Y	Y		[77]
P29401	Transketolase	pentose phosphate-glycolysis	68	ex		Y	Y	[73]
P60174	Triosephosphate isomerase 1	glycolysis	27	ex		Y		[76]
	**CYTOSKELETAL COMPONENTS**							
P60709	Actin, beta	cell growth	42	ex	Y	Y	Y	[70, 71]
P12814	Actinin, alpha 1	cell growth	103	ex	Y	Y		[73, 74]
Q71U36	Alpha-3 tubulin	cell growth	50	ex	Y	Y		[73]
Q14019*	Coactosin-like protein	actin-binding protein	16	In		Y		
P15924	Desmoplakin	cell adhesion	332	ex		Y		[77]
Q16555*	Dihydropyrimidinase-like 2	tubulin binding protein	62	In		Y		
P14923*	Junction plakoglobin	cell adhesion	82	In		Y		
P09382	Lectin, galactoside-binding, soluble, 1 (galectin 1)	growth regulation	15	ex		Y		[21]
P26038	Moesin	cell-matrix interaction	68	ex	Y	Y	Y	[77]
P07737	Profilin 1	cell growth	15	ex		Y	Y	[73]
Q15293*	Reticulocalbin 1, EF-hand calcium binding domain	calcium-binding protein	39	In		Y		
P08670	Vimentin	cell growth	54	ex			Y	[71, 73]
P18206	Vinculin	cell growth	124	ex		Y	Y	[73]
O75083	WD repeat-containing protein 1 isoform 1 variant	actin pol. control	66	ex	Y	Y		[76]
	**HEAT SHOCK/PROTEIN FOLDING PROTEINS**							
P11021	78 kDa glucose-regulated protein precursor (GRP78)	chaperone	72	sp	Y	Y	Y	
O43852	Calumenin precursor (Crocalbin)	calcium-binding chaperone	37	sp	Y	Y	Y	
P27797	Calreticulin	calcium-binding chaperone	48	sp	Y	Y		
P50990*	Chaperonin containing TCP1, subunit 8 (theta)	chaperone	60	In		Y		
P68104	Elongation factor 1-alpha 1 (EF-1-alpha-1)	protein biosynthesis	50	ex		Y		[78]
P11142	Heat shock 70 kDa protein 8 and homologs	chaperone	71	ex		Y		[28]
	Heat shock 70 kDa protein 2 (P54652), Heat shock 70 kDa protein 1-like (P43931)							
P10809	Heat shock 60 kDa protein 1 (chaperonin)	chaperone	61	ex		Y		[29]
P07900	Heat shock protein 90 kDa alpha (cytosolic), class A member 1	chaperone	85	ex		Y		[77]
P30101	PDIA3 protein disulfide-isomerase A3 precursor (EC 5.3.4.1)	protein folding	57	sp	Y	Y	Y	
P23284	Peptidyl-prolyl cis-trans isomerase B precursor, (Rotamase)	protein folding	23	sp	Y	Y	Y	
Q15084	Protein disulfide isomerase family A, member 6	protein folding	48	ex	Y	Y		[71]
P14625	Endoplasmin precursor, (94 kDa glucose-regulated protein) (GRP94) (gp96 homolog) (Tumor rejection antigen 1)	chaperone	92	sp		Y		
	**OTHER PROTEINS**							
P41250	Glycyl-tRNA synthetase (EC 6.1.1.14) (Glycine – tRNA ligase) (GlyRS)	tRNA synthetase	83	sp		Y		
Q9NTK5*	Putative GTP-binding protein 9 (putative)	GTP-binding protein	45	In		Y		
P50395	RAB GDP dissociation inhibitor beta	vesicular transport	51	sp		Y		
P30153*	Serine/threonine-protein phosphatase 2A 65 kDa regulatory subunit A alpha isoform, (Medium tumor antigen-associated 61 kDa protein)	serine/threonine phosphatase	65	In		Y		

Y: indicates the presence of the candidate in the sample; sp: signal peptide; ex: extracellular; in: intracellular; Mw: molecular weight; adipo:day 3 adipocytes; osteo: day 3 osteoblasts; Loc: localisation; ref: reference. * indicates only putatively

**Figure 2:**
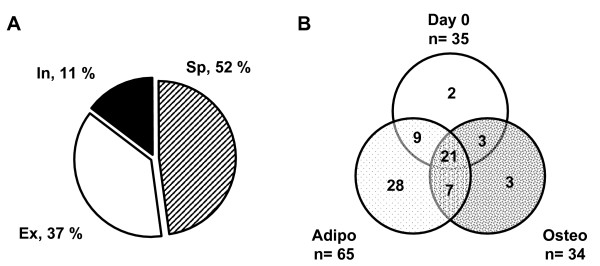
**Protein distribution of hMADS cell secretome**. **A**. hMADS cell secretome is represented using a pie chart in three main groups: secreted proteins with signal peptide (sp, dashed area), secreted proteins without signal peptide (ex, white area) and intracellular proteins (in, dark area). The percentage of the proteins present in each group is reported in the scheme. One hundred per cent is referred to a total number of 73 identified proteins. **B**. Venn diagram of proteins expressed under the different culture conditions; n represents protein number in each condition.

Seventy three proteins were classified according to their function and localization, determined by the Meta search engine Bioinformatic Harvester. As reported in Figure 2A and Table 1, a large amount of identified proteins (52 %) corresponded to "classical" secreted proteins characterized by a signal peptide. Among them, we found several components of the extracellular matrix (fibronectin, collagens etc...), proteases, serine protease inhibitors (serpins such as PAI-1, pigment epithelium derived factor (PEDF) and Plasma protease C1 inhibitor) and other proteins involved in collagen catabolism, protein folding/turnover and growth regulation. In differentiating osteoblasts at day 3, periostin, described previously to be associated with osteoblast differentiation [23] was identified, whereas its presence was not detected in secretion media of differentiating adipocytes. A high percentage of proteins (37 %), devoid of signal peptide, was also detected into the secretion medium. These proteins were labelled as extracellular since they have been previously described in the extracellular compartment. Some of these proteins were metabolic enzymes such as α-enolase (ENO1) and pyruvate kinase, cytoskeletal components or heat shock/chaperone proteins. Similar findings from literature have been reported (see references in Table 1) strengthening actually their presence in the extracellular medium of various cellular models as well as in the murine/human bloodstream. The remaining proteins (11 %) did not exhibit a signal peptide (in: intracellular) and, to the best of our knowledge, have not been so far described extracellularly and did not represent artefacts as cell lysis was very low [see Additional file 1]. These proteins are only putatively secreted and indicated by an asterisk in Table 1. As illustrated by the Venn diagram in Figure 2B, the secretion of 21 proteins resulted to be similar under the three culture conditions, while 28 proteins were specifically secreted under adipogenic condition compared to 3 proteins under osteogenic condition.

**Figure 3:**
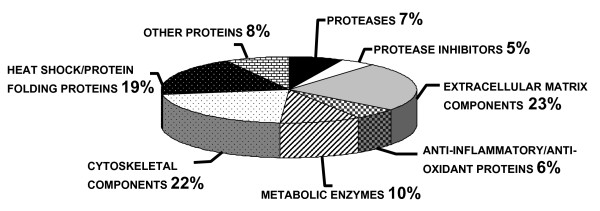
**Distribution into clusters of hMADS cell secretome**. Identified proteins were distributed in 8 main clusters created to classify hMADS cell secreted proteins and are presented in the pie chart. For each cluster the percentage of proteins included is reported.

Finally, hMADS cell secretome was clustered in 8 main groups, as indicated in Table 1 and Figure 3. These clusters include: proteases, protease inhibitors, extracellular matrix (ECM) components, anti-inflammatory/anti-oxidant proteins, metabolic enzymes, cytoskeletal components, heat shock/protein folding proteins and other proteins. These clusters illustrated the diversity of hMADS cell secretome, thus suggesting that some of these proteins could be involved in the regulation of the adipocyte/osteoblast balance.

### Validation of protein expression of selected candidates

**Figure 4:**
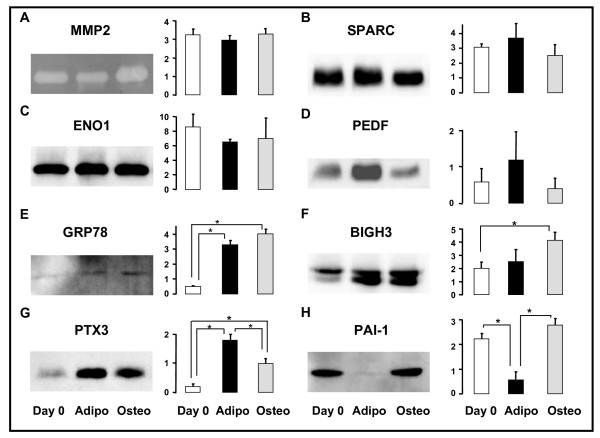
**Secretion levels of 8 candidates released from hMADS cells during the commitment to adipocytes and osteoblasts**. The activity of MMP2 has been evaluated by gelatin zymography (**A**). The expression of SPARC (**B**), ENO1(**C**), PEDF (**D**), GRP78 (**E**), BIGH3 (**F**), PTX3 (**G**) and PAI-1 (**H**) has been analyzed by Western blot. The bar graphs report the levels of expression of every single candidate as the mean of three independent experiments after 6 h of incubation. The values are indicated as arbitrary units. *: p < 0.05. Two μg of secreted proteins have been loaded for each gel.

Mass spectrometry identification data were validated for 8 candidates, selecting them from different clusters, *i.e*. 1 protease, 2 protease inhibitors, 2 ECM components, 1 anti-inflammatory/anti-oxidant protein, 1 metabolic enzyme and 1 heat shock protein. Western blots or zymograms were employed to supplement the identification by mass spectrometry and to determine the expression profile among three different sets of conditions (day 0 and day 3 for adipogenesis or osteogenesis). For all the candidates, the results of immunoblotting/zymogram and a semi-quantification of the bands are reported in Figure 4.

Matrix metallopeptidase 2 (MMP2), secreted protein, acidic, cysteine-rich (SPARC) and ENO1 were secreted by hMADS cells under all conditions, confirming the pattern of detection obtained by SDS-PAGE-mass spectrometry analysis (Fig. 4A–C, to be compared with Table 1). However, no significant difference was observed under the three conditions for these candidates. Zymographic analysis of the secretion media revealed a prominent band of lysis at 66 kDa corresponding to the reported active form of MMP2 (Fig. 4A). Addition of EDTA, an inhibitor of gelatinase activity, led to the absence of lytic bands (data not shown), thus confirming the specificity of MMP activity. Pigment epithelium derived factor (PEDF) was highly expressed in day 3 adipocytes compared to day 0 and day 3 osteoblasts (Fig. 4D) in agreement with mass spectrometry identification.

For 78 kDa glucose-regulated protein precursor (GRP78), transforming growth factor-beta-induced protein (BIGH3), pentraxin 3 (PTX3) and PAI-1, we confirmed the pattern of detection reported in Table 1, with significant differences of expression between day 0, day 3 adipocytes and day 3 osteoblasts, as shown by Western blot and histogram semi-quantifications (Fig. 4E–H).

### Analysis of the plasminogen system in hMADS cells differentiating towards adipocytes and osteoblasts

**Figure 5:**
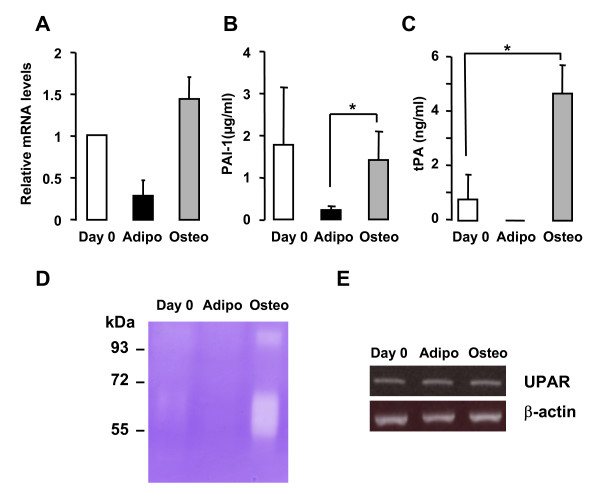
**Evaluation of the presence of the plasminogen system in hMADS cells**. **(A) **PAI-1 mRNA levels have been determined in hMADS cells at day 0, day 3 adipocytes (adipo) and day 3 osteoblasts (osteo). **(B) **PAI-1 and **(C) **tPA protein levels have been measured by ELISA in the secretion media of hMADS cells at day 0, day 3 adipocytes (adipo) and day 3 osteoblasts (osteo) after 6 h of incubation. *: p < 0.05. **(D) **Zymographic analysis of plasminogen activators activity in hMADS cell conditioned media collected after 6 h of incubation. A representative casein-plasminogen zymogram out of three independent experiments is shown. **(E) **RT-PCR analysis of UPAR expression in hMADS cells at day 0, day 3 adipocytes and day 3 osteoblasts. β-actin expression is reported as internal control. PCR products have been separated on a 1% agarose gel.

Given the pattern of expression at the early step of adipogenesis and osteogenesis of hMADS cells, PAI-1 appeared as one of the most promising candidates for investigating further the adipocyte/osteoblast balance. PAI-1 mRNA levels were similar to those of protein levels (Fig. 5A). Since PAI-1 is a key regulator of the conversion of plasminogen to plasmin, negatively acting on two plasminogen activators, namely uPA (urinary plasminogen activator) and tPA (tissue type plasminogen activator) [24], we decided to evaluate uPA and tPA levels in hMADS cell secretome. ELISA experiments were performed on secretion media from hMADS cells at day 0 and day 3 under adipogenic and osteogenic differentiating conditions after 6 h of incubation. As reported in Figure 5B, consistent with the immunoblotting data, PAI-1 levels strongly decreased in differentiating adipocytes as compared to day 0. By contrast, PAI-1 was secreted by day 3 osteoblasts, at albeit the same levels when compared to day 0. Concerning tPA measurement, we did not detect tPA in day 3 differentiating adipocytes, while a significant increase of tPA levels was observed in day 3 osteoblasts as compared to day 0 (Fig. 5C). By contrast, uPA remained undetectable in the secretion media of hMADS cells under the three conditions analyzed.

Enzymatic activities of uPA and tPA in 6 h serum-free conditioned media of hMADS cells at d0 and day 3 of differentiating adipocytes and osteoblasts were then evaluated by casein/plasminogen zymographic analysis. As reported in Figure 5D, tPA was mostly secreted by day 3 osteoblasts, as observed by the lytic band at 63 kDa, while uPA was undetectable (expected band at 48 kDa), thus supporting ELISA results. The higher band at around 110 kDa may correspond to complexes between plasminogen activators and PAI-1 as classically reported [25]. Incubation of the gel with amiloride, which selectively abrogates uPA-dependent enzyme activities, did not affect tPA-dependent lytic bands (data not shown). Furthermore, we demonstrated that hMADS cells expressed uPA receptor (UPAR) mRNA as reported by semi-quantitative RT-PCR analysis (Fig. 5E). As these data actually suggest the involvement of plasminogen activation in the regulation of the fine balance between adipogenesis and osteogenesis, this hypothesis was next examined in a pathophysiological situation where such an imbalance occurs.

### PAI-I expression in ovariectomized mice

**Figure 6:**
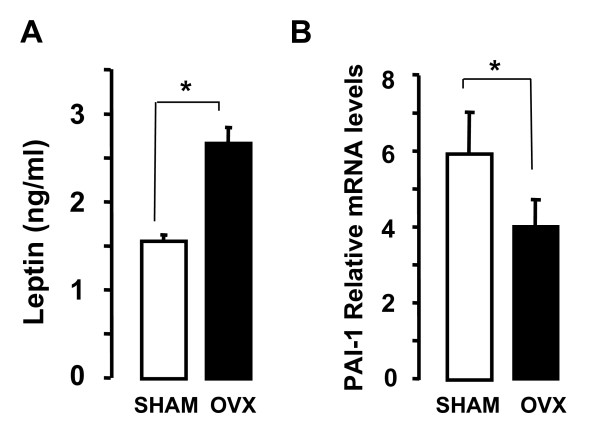
**PAI-1 mRNA levels in the humerus of ovariectomized as compared to control mice**. **A. **Plasma leptin levels have been determined by ELISA. **B. **PAI-1 mRNA expression has been evaluated by qRT-PCR in the humerus of ovx and sham mice and standardized with TBP expression levels. The data are expressed as the mean ± SD *per *group of 4 mice. *: p < 0.05.

Disequilibrium of the adipocyte/osteoblast balance is associated with the development of osteoporosis after ovariectomy. It is well established that in the bone of ovariectomized (ovx) mice, adipocytes develop at expense of bone formation [26,27]. In order to evaluate whether osteoporosis affects PAI-1 in bone, we analyzed the expression of PAI-1 mRNA in the humerus of ovx compared to sham mice. Ten weeks after surgery, as previously described [26] mice increased fat mass as shown by leptin levels (Fig. 6A). As shown in Figure 6B, the levels of PAI-1 mRNA decreased significantly in the humerus of ovx compared to sham mice, thus pointing out at PAI-1 modulation in the adipocyte/osteoblast balance as a potential marker of osteoporosis.

## Discussion

### Characterization of hMADS cell secretome

The secretome of murine and human adipocytes has been characterized [19-22]. However, these studies primarily focused on the late events of adipogenesis. Concerning osteoblast secretome, a single detailed characterization has been performed so far by Xiao and collaborators, who analyzed the extracellular matrix vesicle proteome of mineralizing osteoblasts and identified proteins at late steps of osteogenesis of MC3T3-E1 cells [28].

Herein, proteomic analysis has been used for the first time to compare the secretome of hMADS cells differentiating into adipocytes and osteoblasts and to gain insights into the adipocyte/osteoblast balance. In order to delineate the initial secretory events of adipogenesis and osteogenesis, we performed a 6 h short term secretion which likely limits the number of secreted proteins and cell lysis. A total of 73 proteins was identified and classified into 8 clusters. The largest cluster corresponds to extracellular matrix proteins, thus confirming the secretome characteristic of our fraction. In addition, more than 50% of hMADS cell secretome is represented by proteins with signal peptide. Cytoskeletal proteins and heat shock and folding proteins are also largely represented with 14 and 12 proteins, respectively. Among the heat shock/folding proteins, two chaperones, e.g. HSP60 and HSP70 were found. These findings are reinforced by recent observations describing the presence of HSP60 and HSP70 in the bloodstream. HSP60 circulating levels in diabetic patients have been found associated to cardiovascular diseases [29], while elevated HSP70 circulating levels are related to systemic inflammatory reaction in patients with heart failure following acute myocardial infarction [30]. In the metabolic enzyme cluster, 9 enzymes were also found, including glyceraldehyde dehydrogenase (GAPDH) and phosphoglycerate kinase (PGK). According to literature, extracellular GAPDH, also found in rat serum, inhibits spreading of COS-7 cells [31], while PGK is secreted by tumor cells and its activity is regulated by hypoxia [32]. These enzymes might reflect increased metabolic requirements, but they could also exert independent and still undiscovered functions. Anti-oxidant proteins, like Thioredoxin reductase 1 (TXNRD1), represent another cluster of proteins secreted by hMADS cells. TXNRD1, that does not exhibit a signal peptide, has been described in rat as a circulating protein [33].

Eleven percent of secreted proteins from hMADS cells is however represented by intracellular proteins, presence of which has not been reported up to now outside the cells. A modest contamination by uncontrolled release of intracellular proteins cannot be excluded despite the fact that no cell lysis was detected. Although cell death can take place in culture and is unavoidably accompanied by a subsequent release of cytosolic components, it should be pointed out that cell viability was not affected [see Additional file 1]. Among non-artifactual explanations, it is worth pointing out that this phenomenon could implicate exosome secretion, as most of the identified proteins were reported to be present in exosomes. Exosomes are membrane vesicles that originate from the cell membrane and are released extracellularly [34]. Their occurrence has been also described in the bloodstream and in different tissues *in vivo *[35], suggesting their participation in physiological and/or pathological processes. To date, the exact function of exosomes *in vivo *is not completely understood. A large number of cell types, including reticulocytes, immune cells, platelets, epithelial cells, dendritic cells and others (see [34] and [36] for detailed reviews) appears to secrete exosomes either as a mechanism to extrude some proteins and/or to communicate between cells and/or to play immune functions. Typically, exosomes contain chaperones, cytoskeletal proteins (moesin, desmoplakin), elongation factors and several enzymes (ENO1), as described in literature through proteomic analysis of the secretion media from different sources as well as from analysis by flow cytometry and Western blot [34,37]. Therefore intracellular proteins recovered outside hMADS cells may be a mere reflection of events taking place within the cytosol at the time of exosome formation. Furthermore, 4 (Q14019, Q15293, P50990 and P30153) out of 8 candidates, known to be intracellular, have a score higher than a fixed threshold when analyzed using software predicting a non-classical protein secretion [38], it is likely that these proteins are secreted by non-traditional mechanisms and did not arise from cell lysis. Clearly, these proteins can only be considered as putatively secreted and their actual secretion should be assessed.

### Selected candidates of hMADS cell secretome

Mass spectrometry data have been validated for 8 selected candidates. MMP2, SPARC and ENO1 displayed no significant differences in terms of quantification by immunoblotting or zymogram when comparing the three culture conditions.

MMP2, a zinc-dependent endopeptidase, involved in the degradation of the ECM, is actively secreted by adipocytes [39] and osteoblasts [40]. The presence of MMP2 in hMADS cells reinforces the concept that ECM remodelling represents a crucial event during the early steps of differentiation. As observed in this study, SPARC has been already described as a secreted molecule in both adipocytes [41] and osteoblasts [42]. SPARC is reported to be up-regulated in the adipose tissue of different models of murine obesity [41] and its circulating levels correlate with body mass index in humans [43]. A potential interest in further studying SPARC in the adipocyte/osteoblast balance relies upon the fact that bone marrow stromal cells from SPARC-null mice tend to form more adipocytes than cells from wild type mice [44]. ENO1 is mainly described for its role as an intracellular glycolytic enzyme, however its presence extracellularly has been already reported in the secretion medium of 3T3-L1 adipocytes [21], as well as in other cellular models [45]. ENO1 is a multifunctional protein and a putative plasminogen receptor, since it has been reported as a cell surface protein [46]. Therefore, a potential role of ENO1 in the plasminogen cascade cannot be excluded.

Concerning the five other candidates (PEDF, GRP78, BIGH3, PTX3 and PAI-1), relevant differences of expression between day 0 and day 3 of adipogenesis and osteogenesis have been found. PEDF has been reported to be preferentially secreted by 3T3-L1 preadipocytes as compared to fully differentiated adipocytes [20]. However, Zvonic et al. [22] have described an increase in PEDF expression during adipogenesis of primary cultures of human adipose-derived stem cells, in agreement with our observations in hMADS cells when comparing hMADS cells at day 0 and after complete adipocyte differentiation (data not shown). PEDF could represent a valuable candidate for the adipocyte/osteoblast balance given its expression also in differentiating hMADS osteoblasts. A putative role for PEDF as mediator of angiogenesis and in matrix remodelling of the bone has been recently postulated [47].

Several molecular chaperones have been found in the secretome of hMADS cells, including calumenin, calreticulin, protein disulfide isomerase 6, heat shock proteins and GRP78. As GRP78 controls intracellular protein transport [48], its up-regulation in hMADS differentiating adipocytes and osteoblasts as compared to day 0 could reflect an increased demand in protein folding or a protection against endoplasmic reticulum stress.

BIGH3 is reported to be down-regulated during the differentiation of murine osteoblasts and to play negative effects on the early stages of osteogenesis [49]. In hMADS cells, BIGH3 protein levels increase in differentiating osteoblasts as compared to day 0, however a decrease during osteoblast terminal differentiation was observed (data not shown). Interestingly, BIGH3 is also released by differentiating adipocytes at day 3. So far, no report has described the secretion of BIGH3 by adipocytes or adipose tissue, whereas BIGH3 is involved in distinct cellular functions, such as cell growth, tumorigenesis, wound healing, apoptosis and migration [50]. Thus a role for BIGH3 in adipogenesis remains to be established.

PTX3 is a member of the pentraxin family that we described to be secreted by adipocytes and to respond to inflammatory stimuli such as TNFα [51]. In differentiating hMADS cells, PTX3 appears up-regulated during both adipogenesis and osteogenesis. The presence of PTX3 in osteoblasts has never been reported; we speculate that, under conditions of stress or inflammation, PTX3 could represent a potential target of the adipocyte/osteoblast balance that might deserve further studies.

Finally, PAI-1 represents another member of the serpin family which is secreted by hMADS cells. PAI-1 is a pleiotropic molecule, exerting functional roles in wound healing, atherosclerosis, tumor angiogenesis, rheumatoid arthritis, fibrosis etc., besides its main role as regulator of fibrinolysis [52]. In addition, PAI-1 plays a role in metabolic disorders such as obesity and insulin resistance, representing a marker of metabolic syndrome [53]. A role for PAI-1 in bone remodeling has been postulated, since mice lacking PAI-1 are protected from trabecular bone loss after ovariectomy, suggesting a site-specific role for PAI-1 in bone turnover [54]. Altogether, these data indicate that PAI-1 should represent a reliable candidate for the study of the adipocyte/osteoblast balance. In hMADS cell secretome, PAI-1 signal disappears at day 3 of adipogenesis while remaining present in differentiating osteoblasts. Despite some demonstration that PAI-1 is produced by adipocytes during adipogenesis [53], it was recently shown that adipogenesis, as such, may not induce PAI-1; rather it enhances the potential of adipocytes to respond to PAI-1 inducers [55]. This may explain why PAI-1 synthesis dropped after differentiation of human adipocytes cultured in the absence of serum, known to contain several PAI-1 inducers [56,57]. Moreover overexpression of PAI-1 by adenovirus-mediated gene transfer inhibited adipocyte differentiation [58]. Conversely, preadipocytes from PAI-1^-/- ^mice showed greater differentiation than those issued from wild type mice [58]. This suggests that not only PAI-1 is dispensable for adipocyte differentiation but could even be deleterious as it has been recently suggested that bone marrow-derived PAI-1 had an effect on the development of obesity through its effect in inflammation [59].

tPA is also preferentially secreted by hMADS differentiating osteoblasts, while no detectable levels of uPA have been detected, despite the presence of UPAR mRNA. Interestingly, PAI-1 and tPA serum levels have been reported as putative non-invasive diagnostic biomarkers of idiopathic osteonecrosis of the femoral head [60]. As for circulating PAI-1, a correlation with dysbaric osteonecrosis has been also described [61]. These data strongly suggest that the plasminogen/plasmin system could represent a potential target for further investigation of the adipocyte/osteoblast balance. Even if preliminary, our data concerning a decrease of PAI-1 mRNA levels in bone of ovx as compared to sham mice clearly indicate that PAI-1 expression is modulated under conditions altering the adipocyte/osteoblast balance, such as osteoporosis.

## Conclusion

In conclusion, we have characterized the secretome of hMADS cells during the early events of adipogenesis and osteogenesis using a proteomic approach. hMADS cell secretome is represented by *bona fide *secreted proteins, proteins secreted through non-classical pathways and intracellular proteins, the presence of which in the extracellular medium being likely due to exosome secretion. Eight clusters have been identified, including proteases, protease inhibitors, ECM components, anti-inflammatory-antioxidant proteins, metabolic enzymes, cytoskeletal components, heat-shock/protein folding proteins and other proteins. Among the 8 selected proteins, PAI-1 constitutes the most promising candidate for further studies owing to the role of the plasminogen/plasmin system in adipogenesis and bone remodeling.

## Methods

### Materials

Cell culture media were purchased from Cambrex and FCS from Dutscher S.A. (Brumath, France). EGF was a product of Euromedex (Souffelweyersheim, France). Antibodies directed against ENO1, PEDF, BIGH3 and SPARC were supplied from Santa Cruz Biotechnology (Santa Cruz, CA), Chemicon International (Temecula, CA), R&D Systems (Minneapolis, MN) and Haematologic Technologies Inc. (Essex Junction, VT), respectively. PAI-1 antibody was a kind gift from Prof. PJ Declerck, (Leuven, Belgium). Rat anti-human PTX-3 antibody was kindly provided by Prof. A Mantovani [62]. Reverse transcriptase and trypsin were from Promega (Charbonnières-les-Bains, France). PVDF membranes were from Amersham Biosciences (Orsay, France). Complete protease inhibitor cocktail was from Roche Diagnostics (Meylan, France). All the other products were from Sigma-Aldrich (Saint Quentin Fallavier, France).

### Cell culture

The establishment and characterization of the multipotency and self-renewal of hMADS cells have already been described [14-16]. In the experiments reported herein, hMADS-2 cells, established from the pubic region fat pad of a 5-year old male donor, were used at passages between 16 and 35 corresponding to 35 to 100 population doublings. Cells were seeded at a density of 5000 cells/cm^2 ^in Dulbecco's modified Eagle's medium (DMEM) supplemented with 10% FCS, 2.5 ng/ml hFGF2, 60 μg/ml penicillin and 50 μg/ml streptomycin. The medium was changed every other day and hFGF2 was removed when cells reached confluence. At day 2 post-confluence (designated as day 0) adipogenic or osteogenic differentiation was induced, as described previously [14,17]. Briefly, for adipogenic differentiation hMADS cells were induced to differentiate in the presence of DMEM/Ham's F12 media supplemented with 0.85 μM insulin, 0.2 nM triiodothyronine, 10 μg/ml transferrin, 1 μM dexamethasone (DEX) and 500 μM isobutyl-methylxanthine (IBMX). Three days later, the medium was changed (DEX and IBMX omitted) and 100 nM Rosiglitazone was added up to day 9. For osteogenic differentiation, cells were induced to differentiate in α-MEM containing 1% FCS supplemented with 10 nM 1,25-dihydroxyvitamin D3, 100 nM DEX, 10 μM L-ascorbic acid phosphate, 10 mM β-glycerophosphate and 10 ng/ml EGF. For both differentiation protocols, the media were then changed every other day and cells were used at the indicated days.

Lactate dehydrogenase (LDH) activity has been performed as described previously [63]. Briefly, the activity has been measured in the culture media and compared to that obtained from cells treated with a detergent in order to release the whole intracellular LDH activity.

### Animals

Eight weeks old C57Bl/6J mice were subjected either to bilateral ovariectomies (ovx) from the dorsal approach or to sham surgery during which the ovaries were exteriorized but replaced intact by the operator (Charles River, L'Arbresle, France). Mice were housed in local animal facility for a period of 10 weeks following surgery to await the development of osteoporosis. Animals were sacrificed with carbon dioxide according to the guidelines of the local animal care and experimentation committee. RNA from humerus was extracted using Totally RNA™ kit according to manufacturer's instructions (Ambion, Courtaboeuf, France).

### Sample preparation

Conditioned media for each condition (24 ml, corresponding to 8 dishes of 100 mm diameter) were collected on ice, centrifuged and filtered to remove cell debris, if any, and supplemented with complete protease inhibitor cocktail. Twelve ml of the samples were concentrated by ultra-filtration (Millipore, Centricon, 5 kDa cut-off). Protein concentrations were determined using Bio-Rad Protein Assay reagent (Bio-Rad, Marnes-la-Coquette, France) and were: 0.12 ± 0.04, 0.15 ± 0.06; 0.12 ± 0.02 μg/μl for day 0, day 3 adipocyte and day 3 osteoblast respectively.

### One-dimensional polyacrylamide gel electrophoresis

One-dimensional polyacrylamide gel electrophoresis (SDS-PAGE) was performed using Bio-Rad mini-Protean system. Gels were stained with Bio-Safe Coomassie Stain (Bio-Rad) for 2 h at room temperature and rinsed with water, according to manufacturer's instructions.

### Trypsin digestion and Mass Spectrometry

After Coomassie staining, the whole lanes were manually excised into 10 bands which were processed for tryptic digestion according to a standard protocol. Briefly, after a washing step with 50% ethanol, proteins were reduced and alkylated with 10 mM DTT and 50 mM iodoacetamide, respectively. The spots were washed twice with 50% ethanol, shrunken with acetonitrile (ACN) and then subsequently digested with 100 ng of trypsin in 50 mM ammonium bicarbonate, 15% ACN. The peptides were extracted with 0.5% TFA, 50% ACN then 100% ACN. LC-MS/MS mass spectrometry was performed with a Surveyor system coupled with an LCQ DECA XP ion trap mass spectrometer (ThermoQuest, San Jose, CA). Peptides were separated on a C18 column (300 μm × 10 cm, Hypersil, ThermoQuest) at 5 μl/min with 1 to 40 % ACN gradient in 0.1 % formic acid. A 3 kV voltage was applied on the micro-ESI needle and the automatic acquisition was set as previously described [64].

Fragmentation spectra acquired by Xcalibur 1.3 (ThermoElectron Corp.) were searched with Sequest (Bio-Works version 3.3, Thermo Electron Corp.) against the Swiss-Prot protein database (235 673 entries, uniprot_sprot.fasta file downloaded on October 2006 from [65]). Sequest parameters were: *i*) peptide mass tolerance of 1.7 Da, *ii*) parent ion masses treated as monoisotopic, *iii*) fragmentation ion masses treated as monoisotopic with 1.0 Da mass tolerance, *iv*) trypsin (KR, strictly enzymatic specificity at the both ends) and two miss cleavages, *v*) a 57.0 Da static modification on cysteines accounted for alkylation and a 16.0-Da variable modification on methionine accounted for oxidation.

Sequest results were filtered with the following requirements: *i*) at least 2 peptides were required for protein identification, *ii*) peptides identified as first candidate, *iii*) Xcorr > 1.7, 2.2 and 3.3 for mono-, di- and tri-charged peptides, iv) P value up to 0.001 for peptide. The filters' relevance was checked with different tryptic digests as control (HSP90, albumin, lactoglobulin) and using the different SDS-PAGE molecular weight markers; filters automatically removed all the proteins from Bioworks results sheet with the exception of the exact identification. To address the database redundancy issue, only identifications with highest scoring and corresponding to human protein references were selected. Protein classification has been performed using annotation from Bioinformatic Harvester [66] and Swiss-Prot Protein database [67].

### RNA extraction, RT-PCR and quantitative RT-PCR analysis (qRT-PCR)

Total RNA was isolated with TRI-Reagent kit (Euromedex, France) according to the manufacturer's instructions. Reverse transcriptase reactions and semi-quantitative PCR assays were performed as already described [14,51]. PCR products (25 cycles) were analyzed by 1.5% agarose gel electrophoresis visualized by ethidium-bromide staining.

qRT-PCR assays were run on an ABI Prism 7000 real-time PCR machine (PerkinElmer Life Sciences). Reactions were performed according to manufacturer's instructions using SYBR green master mix (Eurogentec, France). The expression of selected genes was normalized to the expression of the TATA-binding protein (TBP) encoding gene. Gene expression was quantified using the comparative-delta Ct method.

Primer sequences and annealing temperatures are reported in Table 2S [see Additional file 3].

### Immunoblotting

Equal amounts of proteins (2 μg) were denatured, reduced and separated on 12% polyacrylamide-SDS gels. Proteins were transferred in 25 mM Tris, 192 mM glycine and 20% ethanol onto PVDF membrane. Blots were blocked for 30 min with TBS (10 mM Tris-HCl pH 7.5 and 150 mM NaCl) plus 0.1% Tween 20 (TTBS buffer) containing 5% dried milk powder (blocking buffer) and then hybridized in the same buffer with specific primary antibodies (0.2 μg/ml) at 4°C. After overnight incubation, the blots were washed twice in TTBS and incubated 1 hr at room temperature in blocking buffer using the appropriate secondary antibodies (Sigma). After 5 washes in TTBS, immunoreactive proteins were visualized using the ECL chemiluminescence's detection kit (Amersham) according to the manufacturer's instructions.

### Determination of MMP activity by gelatin zymography

Proteins with gelatinolytic activity were identified by electrophoresis in 10% SDS-PAGE containing 1 mg/ml gelatin. Two μg of secretion media were directly loaded on gels under nonreducing conditions. After electrophoresis, proteins were renatured by washing twice with 2.5% Triton X-100 in 50 mM Tris-HCl pH 7.4. The gels were then incubated at 37°C for 16 h in an activation buffer (50 mM Tris-HCl pH 7.4, 5 mM CaCl_2 _and 0.2 M NaCl) and stained with Coomassie Brilliant Blue R-250 (in 40% methanol and 10% acetic acid). Destaining was performed in the same buffer in the absence of the dye. Migration of proteins was compared with that of a prestained molecular weight marker. EDTA (15 mM) was added to the incubation buffer in parallel gels, in order to inhibit cation-dependent enzymatic activities.

### Determination of uPA and tPA activities by casein-plasminogen zymography

Two μg of conditioned media were analyzed on 10% polyacrylamide gels containing 1 mg/ml alpha-casein and 10 μg/ml plasminogen (Sigma), under nonreducing conditions. After the electrophoretic run, gels were rinsed once in 100 mM glycine buffer with 2.5% Triton-X 100 for 45 min and then incubated overnight at 37°C in 100 mM glycine buffer, 15 mM EDTA, pH 8.0. Identical gels were incubated in the above buffers containing 500 μM amiloride, in order to discriminate uPA from tPA bands. Gels were then stained with a 0.25% Coomassie Blue/10% acetic acid/40% methanol solution for 1 hr, followed by destaining in a 40% methanol/10% acetic acid mixture. Caseinolytic activity resulting from plasminogen activation was visualized by white lytic bands on a blue background after conversion of plasminogen to plasmin by uPA and tPA. Molecular weights were calculated from the position of prestained markers that were subjected to electrophoresis in parallel lanes. No lytic bands were observed in plasminogen-free gels.

### ELISA

Supernatant PAI-1 antigen was assayed using ELISA's specific for human PAI-1, as previously described [68]. PAI-1 ELISA detects latent and active forms of human PAI-1 and PAI-1 complexes. The assay is insensitive to PAI-2. The amount of uPA and tPA antigen was measured with the Immunobind uPA ELISA kit (American Diagnostic, Greenwich, CT) and the commercially available kit Asserachrom tPA (Diagnostica Stago, France) according to the instructions of the manufacturer. Inactive and active forms of plasminogen activators are all recognized by the ELISA kit. Plasma concentration of leptin was measured by ELISA using EIA kit (SPI-Bio, France).

### Statistical analysis

Data are expressed as mean values ± standard deviation (SD). qRT-PCR and ELISA data and quantifications of immunoblot/zymogram experiments were analyzed using Student's t-test. Statistical significance was assumed at p level <0.05.

## Abbreviations

BIGH3, transforming growth factor-beta-induced protein; ECM, extracellular matrix; EGF, epidermal growth factor; ENO1, α-enolase; ex, extracellular; GRP78, 78 kDa glucose-regulated protein precursor; hFGF2, human fibroblast growth factor 2; hMADS cells, human multipotent adipose-derived stem cells; in, intracellular; LC-MS/MS, liquid chromatography-tandem mass spectrometry; LDH, lactate dehydrogenase; MMP2, Matrix metallopeptidase 2; ovx, ovariectomized; PAI-1, plasminogen activator inhibitor type 1; PEDF, pigment epithelium derived factor; PTX3, pentraxin 3; sp, signal peptide; SPARC, secreted protein, acidic, cysteine-rich; tPA, tissue type plasminogen activator; uPA, urinary plasminogen activator; UPAR, receptor for urokinase-type plasminogen activator

## Authors' contributions

CC did most of the work, including cell culture, sample media preparation, monodimensional gels, immunoblotting, RNA extraction and qRT-PCR experiments, zymograms and contributed to manuscript preparation. OC contributed to cell culture experiments, sample media and RNA preparation and provided tissues from ovx and sham mice. LN and MS conducted mass spectrometry experiments, peptides identification and database searches; LN also contributed to manuscript preparation. MP performed ELISA assays. GA provided useful suggestions and contributed to manuscript preparation. MCA contributed with reagents, provided useful suggestions and participated to manuscript preparation. CD contributed to data interpretation and manuscript preparation. EA contributed to the experimental design, analysis, interpretation, and manuscript preparation. All authors have read and approved the final manuscript.

## Supplementary Material

Additional file 1 **Figure 1S: LDH activity in media. **LDL activity released in the medium after 6 hours of exposition to serum-free medium (white columns) compared to LDH release by complete cell lysis (black columns) from cells at day 0, day 3 adipodipocyte or osteoblast differentiation. Click here for file

Additional file 2 **Table 1S: List of peptides identified by mass spectrometry for the 73 proteins found in hMADS cell secretome. **The identified proteins are indicated in bold with their corresponding peptides listed under the protein annotation. The different values correspond to parameters produced by Bioworks 3.3 after the data filtering. Click here for file

Additional file 3 Table 2S: List of primer sequences for gene expression analysis by qRT-PCR and RT-PCR. Click here for file
