# Supplementary figures and images for: Characterization of human mesenchymal stem cell secretome at early steps of adipocyte and osteoblast differentiation
Source: BMC Mol Biol. 2008 Feb 26;9:26. doi: 10.1186/1471-2199-9-26 (PMC2279142; doi:10.1186/1471-2199-9-26)

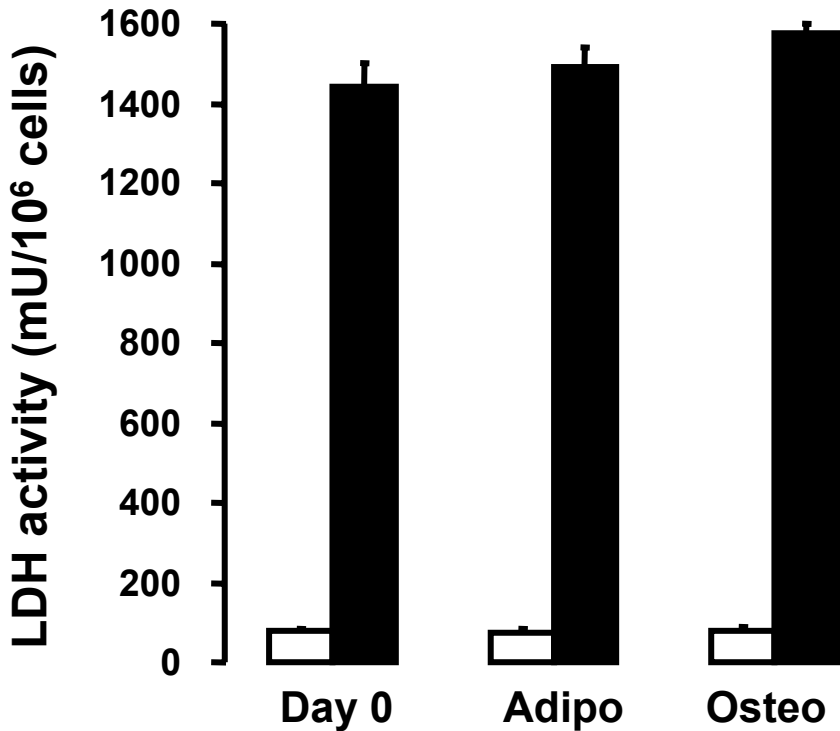

Supplement: Additional file 1 — Figure 1S: LDH activity in media. LDL activity released in the medium after 6 hours of exposition to serum-free medium (white columns) compared to LDH release by complete cell lysis (black columns) from cells at day 0, day 3 adipodipocyte or osteoblast differentiation. [file 1471-2199-9-26-S1.PDF]
