# Supplementary material for: Characterization of human mesenchymal stem cell secretome at early steps of adipocyte and osteoblast differentiation
Source: BMC Mol Biol. 2008 Feb 26;9:26. doi: 10.1186/1471-2199-9-26 (PMC2279142; doi:10.1186/1471-2199-9-26)
Supplement: Additional file 2 — Table 1S: List of peptides identified by mass spectrometry for the 73 proteins found in hMADS cell secretome. The identified proteins are indicated in bold with their corresponding peptides listed under the protein annotation. The different values correspond to parameters produced by Bioworks 3.3 after the data filtering. [file 1471-2199-9-26-S2.PDF]

Table 1S. **Supplementary data. Bioworks 3.3 output sheet for all the identified proteins and the corresponding peptides.**

Peptides/Proteins were automatically filtered with Bioworks 3.3, then exported in the present xls file. The filters and thresholds were:

Xcorr vs Charge State:1.70, 2.20, 3.30

# of Top Matches: 1

Peptide Probability: 0.001

Number of Different Peptides: 2

The Bioworks parameters are described at the end of the table. Bold Characters correspond to protein data, normal characters correspond to peptide data

Reference					P (pro)		Coverage (%)	MW (Da)	Accession	Peptide (Hits)	
	Peptide	MH+	DeltaM	z	P (pep)	XC	DeltaCn				Count
	**PROTEASES**										
**C1R_HUMAN (P00736) Complement C1r subcomponent precursor**					**2,E-13**		**19,57**	**80122**	**P00736**	**9 (9 0 0 0 0)**	
	K.GFLAYYQAVDLDECASR.S	1977,9	-0,4	2	2,E-13	6,46	0,60				
	K.LFGEVTSPLFPK.P	1334,7	0,0	2	2,E-04	3,44	0,44				
	R.LPVANPQACENWLR.G	1667,8	-0,3	2	8,E-13	4,62	0,55				
	K.M*GNFPWQVFTNIHGR.G	1819,9	-0,2	3	2,E-09	4,67	0,58				
	K.QRPPDLDTSSNAVDLLFFTDESGDSR.G	2882,3	0,3	3	2,E-05	4,44	0,25				
	K.TLDEFTIIQNLQPQYQFR.D	2254,2	-0,5	2	3,E-09	4,41	0,56				
	K.VLNYVDWIK.K	1149,6	-0,5	2	3,E-06	3,26	0,44				
	R.WILTAAHTLYPK.E	1413,8	-0,3	2	4,E-07	3,96	0,45				
	R.WVATGIVSWGIGCSR.G	1648,8	-0,3	2	8,E-08	5,02	0,58				
**C1S_HUMAN (P09871) Complement C1s subcomponent precursor**					**2,E-13**		**19,48**	**76635**	**P09871**	**9 (9 0 0 0 0)**	
	R.DVVQITCLDGFEVVEGR.V	1935,9	-0,4	2	2,E-13	5,84	0,62				
	K.GFQVVVTLR.R	1018,6	-0,2	2	2,E-04	4,03	0,28				
	K.M*LTPEHVFIHPGWK.L	1707,9	0,0	3	7,E-05	3,83	0,46				
	K.SNALDIIFQTDLTGQK.K	1763,9	-0,4	2	3,E-12	5,34	0,48				
	R.SSNNPHSPIVEEFQVPYNK.L	2186,1	-0,2	3	6,E-09	4,65	0,39				
	R.TNFDNDIALVR.L	1277,6	-0,3	2	4,E-08	3,85	0,43				
	K.VEDPESTLFGSVIR.Y	1548,8	-0,3	2	2,E-09	4,52	0,49				
	K.VEKPTADAEAYVFTPNMICAGGEK.G	2598,2	0,0	3	8,E-06	4,53	0,19				
	R.VKNYVDWIM*K.T	1311,7	-0,3	2	3,E-05	3,37	0,45				
**MMP1_HUMAN (P03956) Interstitial collagenase precursor (EC 3.4.24.7) (Matrix metalloprotein**					**3,E-12**		**18,34**	**53973**	**P03956**	**6 (6 0 0 0 0)**	
	K.AFQLWSNVTPLTFTK.V	1752,9	-0,5	2	1,E-08	4,40	0,56				
	K.DGFFYFFHGTR.Q	1393,6	-0,8	2	4,E-07	3,80	0,60				
	K.DIYSSFGFPR.T	1188,6	-0,3	2	3,E-06	4,03	0,62				
	K.LTFDAITTIR.G	1150,6	-0,3	2	6,E-07	3,78	0,42				
	K.VSEGQADIM*ISFVR.G	1567,8	-0,3	2	3,E-11	4,57	0,48				
	K.YWAVQGQNVLHGYPK.D	1759,9	-0,3	2	3,E-12	4,83	0,60				
**MMP2_HUMAN (P08253) 72 kDa type IV col**					**7,E-13**		**45,25**	**73835**	**P08253**	**13 (13 0 0 0 0)**	
	R.IHDGEADIM*INFGR.W	1603,8	0,2	3	3,E-04	3,91	0,41				
	K.QDIVFDGIAQIR.G	1374,7	-0,3	2	3,E-09	4,23	0,49				
	K.FPFLFNGK.E	969,5	-0,3	2	4,E-04	2,99	0,45				
	R.AFQVWSDVTPLR.F	1418,7	-0,3	2	1,E-07	4,61	0,39				

K.FPPLFNGK.E	969,5	0,4	2	8,E-04	2,40	0,36			
K.FFGLPQTGDLDQNTIETM*R.K	2199,0	-0,4	2	1,E-09	4,52	0,65			
K.QDIVFDGIAQIR.G	1374,7	-0,3	2	1,E-09	4,41	0,45			
K.FPPLFNGK.E	969,5	-0,3	2	2,E-04	2,77	0,48			
R.IHDGEADIMINFGR.W	1587,8	0,1	3	4,E-05	3,60	0,37			
R.AFQVWSDVTPLR.F	1418,7	-0,3	2	4,E-06	4,01	0,35			
R.IIGYTPDLDPETVDDAFAR.A	2108,0	-0,4	2	7,E-13	4,94	0,61			
K.FFGLPQTGDLDQNTIETMR.K	2183,0	-0,4	2	1,E-12	4,66	0,62			
K.AVFFAGNEYWIYSASTLER.G	2224,1	-0,4	2	1,E-10	5,64	0,53			
**PCOC1_HUMAN (Q15113) Procollagen C-endopeptidase enhancer 1 pre**	**20401,2**			**5,E-12**		**24,94**	**47942**	**Q15113**	**7 (7 0 0 0 0)**
R.EPGEGLAVTVSLIGAYK.T	1703,9	-0,2	2	3,E-07	3,63	0,58			
R.FCGTFRPAPLVAPGNQVTLR.M	2201,2	-0,1	3	1,E-06	4,23	0,47			
R.GFLLWYSGR.A	1098,6	-0,3	2	1,E-04	3,50	0,46			
K.TGGLDLPSPPTGASLK.F	1510,8	-0,3	2	9,E-07	3,06	0,36			
R.TGTLQSNFCASSLVVTATVK.S	2084,1	-0,4	2	1,E-05	3,38	0,42			
R.YDALEVFAGSGTSGQR.L	1657,8	-0,3	2	5,E-12	5,22	0,57			
R.YDSVSVFNGAVSDDSR.R	1717,8	-0,5	2	3,E-11	4,13	0,54			
**PROTEASE INHIBITORS**									
**PAI1_HUMAN (P05121) Plasminogen activator inhibitor 1 precursor (PAI-1)**				**1,E-11**		**18,49**	**45031**	**P05121**	**9 (9 0 0 0 0)**
K.DEISTTDAIFVQR.D	1494,7	0,4	2	5,E-05	2,51	0,28			
K.ELM*GPWNKDEISTTDAIFVQR.D	2466,2	-0,2	3	1,E-07	4,19	0,48			
K.FSLETEVDLR.K	1208,6	-0,2	2	8,E-07	4,08	0,52			
K.IEVNESGTVASSSTAVIVSAR.M	2077,1	-0,5	2	1,E-11	5,87	0,58			
R.KPLENLGM*TDMFR.Q	1567,8	0,5	2	2,E-05	4,20	0,05			
R.KPLENLGMTDM*FR.Q	1567,8	0,3	3	2,E-05	3,72	0,34			
R.LVLVNALYFNGQWK.T	1664,9	-0,4	2	6,E-10	5,84	0,08			
K.LVQGFM*PHFFR.L	1394,7	-0,2	2	3,E-06	3,11	0,44			
K.VKIEVNESGTVASSSTAVIVSAR.M	2304,2	-0,3	3	3,E-05	5,22	0,61			
**PEDF_HUMAN (P36955) Pigment epithelium-derived factor precursor**				**1,E-12**		**34,21**	**46313**	**P36955**	**5 (5 0 0 0 0)**
R.ALYYDLISSPDIHGTYK.E	1956,0	-0,4	2	3,E-10	5,24	0,47			
R.DTDTGALLFIGK.I	1250,7	-0,3	2	3,E-07	5,04	0,47			
K.EIPDEISILLLGVAHFK.G	1894,1	-0,5	2	1,E-11	5,52	0,57			
K.ELLDTVTAPQK.N	1214,7	-0,5	2	2,E-04	3,21	0,25			
K.IAQLPLTGSM*SIIFFLPLK.V	2105,2	0,4	2	1,E-12	4,22	0,52			
**IC1_HUMAN (P05155) Plasma protease C1 inhibitor precursor**				**3,E-11**		**8,92**	**55119**	**P05155**	**3 (3 0 0 0 0)**
K.FQPTLLTLPR.I	1185,7	-0,3	2	5,E-04	2,55	0,24			
R.LLDSLPSDTRLVLLNAIYLSAK.W	2415,4	-0,2	3	3,E-11	4,40	0,60			
R.LVLLNAIYLSAK.W	1317,8	-0,7	2	1,E-06	3,97	0,50			
**TIMP1_HUMAN (P01033) Metalloproteinase inhibitor 1 precursor**				**7,E-14**		**21,19**	**23156**	**P01033**	**5 (5 0 0 0 0)**
R.FVYTPAMESVCGYFHR.S	1963,9	-0,2	2	7,E-14	4,15	0,57			
R.FVYTPAM*ESVCGYFHR.S	1979,9	-0,4	2	4,E-12	3,93	0,50			
K.GFQALGDAADIR.F	1233,6	-0,2	2	3,E-07	4,59	0,42			
K.LQDGLLHITTCSFVAPWNSLSLAQR.R	2827,5	0,5	3	7,E-05	5,00	0,48			
K.LQSGTHCLWTDQLLQGSEK.G	2201,1	0,6	3	2,E-06	3,43	0,39			

**EXTRACELLULAR MATRIX COMPONENTS**									
**SERPH_HUMAN (P50454) Serpin H1 precursor (Collagen-binding protein) (Colligin) (47 kDa he**				**2,E-09**		**12,44**	**46411**	**P50454**	**4 (4 0 0 0 0)**
K.GVVEVTHDLQK.H	1224,7	-0,2	2	9,E-05	2,40	0,38			
R.SAGLAFSLYQAM*AK.D	1473,7	-0,8	2	2,E-09	4,00	0,65			
R.DTQSGSLLFIGR.L	1293,7	-0,8	2	1,E-06	3,14	0,41			
R.LYGPSSVSFADDFVR.S	1659,8	0,1	2	9,E-08	2,88	0,48			
**PGBM_HUMAN (P98160) Basement membrane-specific heparan sulfate proteoglycan core pro**				**1,E-12**		**3,85**	**468528**	**P98160**	**4 (4 0 0 0 0)**
K.DFISLGLQDGHLVFR.Y	1716,9	-0,3	2	3,E-12	4,80	0,50			
R.GSIQVDGEELVSGR.S	1445,7	-0,3	2	1,E-09	4,50	0,50			
K.GSVYIGGAPDVATLTGGR.F	1690,9	-0,3	2	5,E-12	5,38	0,50			
R.SLPEVPETIELEVR.T	1610,9	-0,4	2	1,E-10	2,38	0,42			
**CO1A1_HUMAN (P02452) Collagen alpha-1**				**3,E-12**		**19,16**	**138799**	**P02452**	**8 (8 0 0 0 0)**
K.ALLLKGSNEIEIR.A	1455,9	-0,6	2	2,E-09	3,71	0,50			
R.DLEVDTTLK.S	1033,5	-0,3	2	3,E-04	2,47	0,27			
R.DRDLEVDTTLK.S	1304,7	-0,1	2	8,E-08	3,34	0,34			
R.FTYSVTVDGCTSHTGAWGK.T	2073,9	0,3	3	3,E-08	3,40	0,36			
K.SGEYWIDPNQGCNLDAIK.V	2079,9	-0,4	2	4,E-10	5,05	0,49			
K.SLSQQIENIR.S	1187,6	-0,4	2	5,E-05	3,28	0,24			
K.STGGISVPGPM*GPSGPR.G	1569,8	-0,6	2	4,E-04	2,56	0,34			
K.VLCDDVICDETK.N	1466,7	-0,5	2	2,E-08	3,50	0,65			
**CO1A2_HUMAN (P08123) Collagen alpha-2(I) chain precursor**				**6,E-16**		**23,57**	**129334**	**P08123**	**10 (10 0 0 0 0)**
K.AVILQGSNDVELVAEGNSR.F	1971,0	-0,5	2	6,E-16	5,59	0,57			
K.EMATQLAFMR.L	1197,6	-0,3	2	9,E-07	3,00	0,40			
K.EMATQLAFM*R.L	1213,6	-0,1	2	3,E-05	2,99	0,41			
R.FTYTVLVDGCSK.K	1389,7	-0,3	2	2,E-10	4,20	0,57			
R.FTYTVLVDGCSKK.T	1517,8	0,1	2	5,E-06	3,12	0,38			
K.HVWLGETINAGSQFEYNVEGVTSK.E	2665,3	-0,2	3	4,E-12	5,32	0,55			
K.KAVILQGSNDVELVAEGNSR.F	2099,1	0,2	3	2,E-06	3,53	0,44			
R.LSHPEWSSGYYWIDPNQGCTM*EAIK.V	2985,3	0,6	3	1,E-04	4,46	0,39			
K.NSIAYMDEETGNLKK.A	1712,8	0,8	2	8,E-05	3,40	0,47			
K.SLNNQIETLLTPEGSR.K	1771,9	-0,4	2	4,E-04	2,95	0,47			
**CO3A1_HUMAN (P02461) Collagen alpha-1**				**2,E-11**		**5,25**	**138470**	**P02461**	**7 (7 0 0 0 0)**
K.AGGFAPYYGDEPM*DFK.I	1780,8	-0,4	2	4,E-05	2,38	0,43			
K.FTYTVLEDGCTK.H	1433,7	-0,4	2	2,E-09	4,41	0,54			
K.INTDEIM*TSLK.S	1280,6	-0,4	2	2,E-05	3,16	0,47			
K.INTDEIMTSLK.S	1264,6	-0,9	2	2,E-04	2,86	0,49			
K.NSIAYM*DQASGNVK.K	1513,7	-0,3	2	2,E-07	3,65	0,50			
K.SVNGQIESLISPDGSR.K	1658,8	1,3	2	1,E-05	3,62	0,45			
K.VFCNM*ETGETCISANPLNVPR.K	2425,1	-0,2	3	6,E-05	3,73	0,39			
**CO6A1_HUMAN (P12109) Collagen alpha-1(VI) chain precursor**				**2,E-12**		**8,33**	**108480**	**P12109**	**9 (9 0 0 0 0)**
R.AGIEIFVVVVGR.Q	1258,8	-0,4	2	3,E-06	4,79	0,42			
R.GPEGPQGPPGHQGPPGPDECEILDIIM*K.M	2938,4	0,5	3	4,E-07	4,76	0,51			
K.GYPGLKGDEGEAGDPGDDNNDIAPR.G	2529,1	0,0	3	2,E-09	3,86	0,40			
R.IALVITDGR.S	957,6	-0,3	2	4,E-05	3,48	0,47			

R.LLLFSDGNSQGATPAAIEK.A	1932,0	-0,4	2	5,E-12	6,23	0,53			
R.NLVWNAGALHYSDEVEIIQGLTR.M	2598,3	-0,1	3	2,E-12	4,70	0,34			
K.NNVEQVCCSFECQPAR.G	1997,8	1,4	2	5,E-04	2,88	0,31			
K.VFSVAITPDHLEPR.L	1580,8	0,2	2	1,E-05	2,75	0,44			
R.VPSYQALLR.G	1046,6	-0,1	2	1,E-07	2,84	0,40			
**CO6A2_HUMAN (P12110) Collagen alpha-2(VI) chain precursor**				**5,E-11**		**5,48**	**108506**	**P12110**	**6 (6 0 0 0 0)**
R.DVTVTAIGIGDM*FHEK.H	1748,9	0,3	2	6,E-06	3,51	0,50			
K.DYDSLAQPGFFDR.F	1530,7	-0,2	2	6,E-07	4,24	0,51			
R.LFAVAPNQNLK.E	1214,7	-0,5	2	2,E-04	3,60	0,34			
K.NLEWIAGGTWTPSALK.F	1743,9	-0,6	2	2,E-04	3,65	0,45			
R.PVDIVFLLDGSER.L	1459,8	-0,3	2	5,E-11	4,52	0,61			
R.YGGLHFSDQVEVFSPPGSDR.A	2194,0	0,4	3	2,E-04	4,35	0,30			
**CO6A3_HUMAN (P12111) Collagen alpha-3(VI) chain precursor**				**1,E-11**		**2,33**	**343337**	**P12111**	**8 (8 0 0 0 0)**
R.AAPLQGM*LPGLLAPLR.T	1633,9	-0,4	2	3,E-05	3,00	0,48			
K.DVVFLLDGSEGVR.S	1405,7	-0,4	2	2,E-05	3,40	0,32			
R.EVQVFEITENSAK.L	1493,7	-0,9	2	4,E-04	2,80	0,59			
R.HIVLKPPTIVTQVIEVNK.R	2028,2	0,9	3	3,E-09	4,73	0,56			
K.ISLSPEYVFSVSTFR.E	1731,9	-0,3	2	6,E-09	3,88	0,49			
K.LLTPITTLTSEQIQK.L	1686,0	-0,5	2	2,E-08	4,02	0,58			
K.LSDAGITPLFLTR.Q	1403,8	-0,3	2	8,E-07	4,94	0,53			
R.VAVVTYNNEVTTEIR.F	1707,9	-0,3	2	8,E-08	4,96	0,55			
**PGS2_HUMAN (P07585) Decorin precursor (Bone proteoglycan II)**				**2,E-12**		**10,86**	**39722**	**P07585**	**6 (6 0 0 0 0)**
R.DFEPSLGPVCPFR.C	1520,7	-0,3	2	7,E-07	3,67	0,60			
K.DLPPDTTLLDLQNNK.I	1696,9	-0,8	2	2,E-12	2,23	0,53			
R.KVTFNGLNQM*IVIELGTNPLK.S	2345,3	0,2	3	4,E-04	3,68	0,24			
K.NLHALILVNNK.I	1248,7	-0,7	2	1,E-05	3,12	0,47			
K.VTFNGLNQM*IVIELGTNPLK.S	2217,2	-0,3	2	5,E-12	4,77	0,53			
R.VVQCSDLGLDKVPK.D	1557,8	-0,5	2	3,E-06	3,66	0,40			
**FINC_HUMAN (P02751) Fibronectin precursor**				**1,E-30**		**23,76**	**262440**	**P02751**	**51 (51 0 0 0 0)**
K.DDKESVPISDTIIPAVPPPTDLR.F	2475,3	-0,4	3	3,E-05	4,03	0,46			
R.DLEVVAATPTSLLISWDAPAVTVR.Y	2524,4	-0,6	2	4,E-08	5,29	0,65			
R.DLQFVEVTDVK.V	1292,7	-0,3	2	1,E-05	5,12	0,49			
K.DSM*IWDCTCIGAGR.G	1657,7	-0,4	2	3,E-08	4,49	0,51			
K.EATIPGHLNSYTIK.G	1543,8	0,3	2	8,E-09	2,42	0,40			
R.EESPLLIGQQSTVSDVPR.D	1955,0	-0,3	2	7,E-11	4,53	0,55			
K.EINLAPDSSSVVVSGLM*VATK.Y	2133,1	0,1	2	3,E-11	4,79	0,62			
K.ESVPISDTIIPAVPPPTDLR.F	2117,1	-0,7	2	6,E-05	2,25	0,16			
R.FLATTPNSLLVSWQPPR.A	1927,0	-0,5	2	5,E-12	3,92	0,59			
R.GATYNIIVEALK.D	1291,7	-0,9	2	2,E-05	3,57	0,33			
R.GATYNIIVEALKDQQR.H	1819,0	-0,5	2	6,E-09	4,32	0,40			
R.GDSPASSKPISINYR.T	1591,8	0,0	2	5,E-08	3,18	0,47			
K.GEWTCKPIAEK.C	1318,6	-0,4	2	7,E-05	3,64	0,39			
R.GFNCESKPEAEETCFDKYTGNTYR.V	2903,2	0,7	3	4,E-13	4,54	0,43			
K.GLAFTDVDVDSIK.I	1379,7	-0,2	2	1,E-07	4,47	0,47			

K.GLKPGVVYEGQLISIQQYGHQEVTR.F	2799,5	0,3	3	1,E-10	4,30	0,53			
R.HTSVQTTSSGSGPFTDVR.A	1863,9	-0,4	2	1,E-13	5,18	0,57			
K.HYQINQQWER.T	1401,7	-0,1	2	6,E-05	3,13	0,37			
K.IAWESPQGQVSR.Y	1357,7	-0,2	2	5,E-06	4,24	0,41			
R.ITGYIIKYEKPGSPPR.E	1819,0	0,1	3	6,E-09	3,88	0,55			
R.ITYGETGGNSPVQEFTVPGSK.S	2168,1	-0,4	2	2,E-10	4,82	0,61			
K.IYLYTLNDNAR.S	1355,7	-0,5	2	7,E-06	2,98	0,35			
K.LGVRPSQGGEAPR.E	1323,7	-0,4	3	7,E-07	3,64	0,25			
K.LLCQCLGFGSGHFR.C	1651,8	0,1	3	6,E-07	3,67	0,38			
R.NLQPASEYTVSLVAIK.G	1732,9	-0,5	2	1,E-09	3,28	0,48			
R.NSITLTNLTPGTEYVVSIVALNGR.E	2532,4	-0,3	3	8,E-08	3,99	0,43			
R.NTFAEVTGLSPGVTYYFK.V	1994,0	-0,3	2	4,E-14	5,03	0,62			
R.PAQGVVTTLENVSPPR.R	1664,9	-0,3	2	7,E-05	4,19	0,62			
R.PAQGVVTTLENVSPPRR.A	1821,0	-0,2	3	9,E-05	3,42	0,50			
R.RPGGEPSPEGTTGQSYNQYSQR.Y	2396,1	0,0	3	1,E-09	6,02	0,57			
R.RPHETGGYM*LECVCLGNGK.G	2194,0	0,0	3	2,E-08	4,89	0,49			
R.SSPVVIDASTAIDAPSNLR.F	1913,0	-0,2	3	1,E-06	4,31	0,44			
K.STATISGLKPGVDYTITVYAVTGR.G	2470,3	-0,1	3	3,E-07	4,93	0,57			
R.SYTITGLQPGTDYK.I	1543,8	0,7	2	8,E-07	2,66	0,25			
R.TEIDKPSQMQVTDVQDNSISVK.W	2462,2	-0,1	3	6,E-14	6,31	0,45			
R.TEIDKPSQM*QVTDVQDNSISVK.W	2478,2	0,0	3	1,E-30	6,41	0,45			
R.TFYSCTTEGR.Q	1221,5	-0,4	2	3,E-07	2,95	0,56			
K.TGLDSPTGIDFSDITANSFTVHWIAPR.A	2918,4	0,5	3	3,E-13	4,76	0,40			
R.TKTETITGFQVDAVPANGQTPIQR.T	2572,3	-0,3	3	2,E-04	4,19	0,43			
K.TYHVGEQWQK.E	1275,6	-0,1	2	2,E-06	3,32	0,42			
R.TYLGNALVCTCYGGSR.G	1791,8	-0,4	2	1,E-07	4,50	0,51			
R.VDVIPVNLPGEHGQR.L	1629,9	-0,4	2	2,E-12	3,45	0,49			
R.VPGTSTSATLTGLTR.G	1461,8	-0,5	2	2,E-07	3,30	0,60			
R.VTDATETTITISWR.T	1593,8	-0,1	2	2,E-06	4,43	0,46			
K.VTIM*WTPPESAVTGYR.V	1823,9	0,2	2	3,E-09	3,49	0,52			
R.VTWAPPPSIDLTNFLVR.Y	1926,0	-0,4	2	9,E-13	3,13	0,37			
K.WCGTTQNYDADQK.F	1586,7	-0,3	2	1,E-09	4,32	0,52			
R.WKEATIPGHLNSYTIK.G	1858,0	-0,2	3	2,E-05	3,39	0,44			
K.WLPSSSPVTGYR.V	1349,7	-1,2	2	4,E-05	2,32	0,34			
R.WSRPQAPITGYR.I	1431,7	-0,4	3	3,E-07	3,60	0,32			
K.YEVSVYALK.D	1071,6	-0,2	2	8,E-05	2,57	0,48			
**LG3BP_HUMAN (Q08380) Galectin-3-binding protein precursor**				**7,E-15**		**9,20**	**65289**	**Q08380**	**6 (6 0 0 0 0)**
R.ELSEALGQIFDSQR.G	1592,8	-0,3	2	1,E-06	4,39	0,51			
R.IYTSPTWSAFVTDSSWSAR.K	2162,0	-0,3	2	7,E-15	4,65	0,60			
R.RIDITLSSVK.C	1131,7	-0,3	2	8,E-05	3,57	0,35			
R.SDLAVPSELALLK.A	1355,8	-0,3	2	6,E-07	2,30	0,27			
K.TLQALEFHTVPFQLLAR.Y	1984,1	0,5	3	8,E-09	4,27	0,41			
K.YSSDYFQAPSDYR.Y	1598,7	-0,3	2	4,E-11	4,50	0,57			
**IBP7_HUMAN (Q16270) Insulin-like growth factor-binding protein 7 precursor**				**6,E-09**		**10,24**	**29111**	**Q16270**	**3 (3 0 0 0 0)**

R.GGPEKHEVTGWVLVSPLSK.E	2020,1	0,2	3	2,E-06	4,14	0,53			
K.HEVTGWVLVSPLSK.E	1551,9	-0,3	2	6,E-09	4,42	0,58			
K.ITVVDALHEIPVK.K	1433,8	-0,3	2	3,E-08	4,20	0,51			
**LAMC1_HUMAN (P11047) Laminin gamma-1 chain precursor (Laminin B2 chain)**				**2,E-12**		**1,58**	**177492**	**P11047**	**2 (2 0 0 0 0)**
R.LSAEDLVLEGAGLR.V	1442,8	-0,3	2	2,E-12	5,22	0,43			
R.SAGYLDDVTLASAR.P	1438,7	-0,4	2	1,E-06	4,11	0,53			
**LUM_HUMAN (P51884) Lumican precursor (Keratan sulfate proteoglycan lumican) (KSPG lumi**				**5,E-11**		**18,05**	**38405**	**P51884**	**5 (5 0 0 0 0)**
R.FNALQYLR.L	1024,6	-0,6	2	5,E-04	2,33	0,25			
K.ISNIPDEYFK.R	1225,6	-0,6	2	3,E-04	2,97	0,35			
R.LPSGLPVSLLTLYLDNNK.I	1957,1	-0,7	2	5,E-11	4,60	0,42			
K.SLEDLQLTHNK.I	1297,7	-0,9	2	2,E-04	2,55	0,42			
K.SLEYLDLSFNQIAR.L	1668,9	-0,7	2	2,E-04	3,45	0,35			
**NID1_HUMAN (P14543) Nidogen-1 precursor**				**1,E-11**		**11,79**	**136367**	**P14543**	**19 (19 0 0 0 0)**
K.AFLHVPAKVIIGLAFDCVDK.M	2213,2	0,0	3	1,E-04	3,37	0,43			
K.ALEGLQYPFAVTSYGK.N	1743,9	-0,3	2	3,E-11	4,65	0,56			
K.ESHPGLFPPTFGAVAPFLADLDTTDGLGK.V	2970,5	0,2	3	6,E-10	5,16	0,59			
R.FSGIDEHGHLTIDTELEGR.V	2126,0	0,1	3	3,E-11	5,89	0,54			
R.FYDRSDIDAVYVTTNGIIATSEPPAK.E	2843,4	0,4	3	3,E-06	3,89	0,42			
R.KALEGLQYPFAVTSYGK.N	1872,0	-0,3	3	7,E-06	4,76	0,53			
K.M*NSVVALDLAISK.E	1376,7	-0,3	2	1,E-08	4,71	0,50			
K.MNSVVALDLAISK.E	1360,8	-0,3	2	1,E-09	4,41	0,46			
K.MVYWTDITEPSIGR.A	1667,8	-0,2	2	5,E-09	4,76	0,57			
K.M*VYWTDITEPSIGR.A	1683,8	-0,3	2	5,E-10	4,80	0,57			
K.NGFSITGGEFTR.Q	1285,6	-0,3	2	6,E-06	3,13	0,36			
R.NIFWTDSNLDRIEVAK.L	1921,0	-0,3	2	3,E-10	5,01	0,54			
K.NLYFTDWK.M	1086,5	-0,6	2	2,E-05	2,43	0,41			
R.RVLFETDLVNPR.G	1458,8	-0,2	2	7,E-08	4,37	0,55			
R.SDIDAVYVTTNGIIATSEPPAK.E	2262,1	-0,7	2	1,E-11	4,31	0,65			
R.TQFTCECSIGFR.G	1505,7	-0,3	2	6,E-09	4,04	0,52			
K.VIIGLAFDCVDK.M	1349,7	-0,3	2	6,E-08	4,91	0,60			
R.VLFETDLVNPR.G	1302,7	-0,3	2	9,E-08	4,25	0,54			
K.VYYREDLSPSITQR.A	1726,9	-0,3	2	1,E-08	4,58	0,48			
**POSTN_HUMAN (Q15063) Periostin precursor (PN) (Osteoblast-specific factor 2)**				**9,E-10**		**4,29**	**93255**	**Q15063**	**2 (2 0 0 0 0)**
R.AAAITSDILEALGR.D	1400,8	-0,4	2	1,E-08	5,24	0,57			
K.GCPAVLPIDHVYGTLGIVGATTTQR.Y	2596,4	-0,2	3	9,E-10	3,60	0,32			
**PLOD1_HUMAN (Q02809) Procollagen-lysine,2-oxoglutarate 5-dioxygenase 1 precursor**				**2,E-08**		**9,15**	**83498**	**Q02809**	**5 (5 0 0 0 0)**
K.FLLEYIAPM*TEK.L	1470,8	-0,8	2	5,E-07	4,02	0,42			
R.IFQNLDGALDEVVLK.F	1673,9	-0,4	2	2,E-08	4,91	0,27			
K.LQLNYLGNYIPR.F	1463,8	-0,6	2	7,E-06	3,59	0,50			
R.SEDYVDIVQGR.R	1280,6	-0,7	2	9,E-05	3,63	0,53			
R.SQVVFSAEELIYPDR.R	1752,9	-0,8	2	1,E-04	2,38	0,43			
**QSCN6_HUMAN (O00391) Sulfhydryl oxidase 1 precursor (quiescin)**				**2,E-11**		**14,31**	**82526**	**O00391**	**7 (7 0 0 0 0)**
K.AHFSPSNIILDFPAAGSAAR.R	2042,0	-0,5	3	6,E-08	4,93	0,45			
K.IYM*ADLESALHYILR.I	1823,9	0,1	3	1,E-10	3,38	0,51			

R.LAGAPSEDPQFPK.V	1356,7	-0,1	2	3,E-06	3,20	0,34			
R.LDVPVWDVEATLNFLK.A	1859,0	-0,5	2	2,E-10	4,06	0,62			
K.LEEIDGFFAR.N	1196,6	-0,8	2	5,E-04	3,09	0,39			
R.NNEEYLALIFEK.G	1482,7	-0,6	2	5,E-06	3,37	0,50			
R.SALYSPSDPLTLLQADTVR.G	2047,1	0,9	2	4,E-04	2,45	0,36			
**SPRC_HUMAN (P09486) SPARC precursor (Secreted protein acidic and rich in cyst**				**2,E-13**		**10,56**	**34610**	**P09486**	**7 (7 0 0 0 0)**
R.FFETCDLDNDK.Y	1403,6	-0,6	2	5,E-08	3,75	0,57			
R.LEAGDHPVELLAR.D	1419,8	-0,3	2	1,E-09	4,23	0,41			
K.LHLDYIGPCK.Y	1215,6	-0,3	2	5,E-05	3,22	0,26			
K.NVLVTLYER.D	1106,6	-0,2	2	2,E-04	3,69	0,25			
K.NVLVTLYERDEDNNLLTEK.Q	2278,2	-0,3	2	2,E-13	4,91	0,40			
K.RLEAGDHPVELLAR.D	1575,9	-0,2	2	8,E-09	4,31	0,42			
K.TFDSSCHFFATK.C	1447,6	-0,3	2	3,E-09	4,23	0,54			
**BGH3_HUMAN (Q15582) Transforming growth factor-beta-induced protein ig-h3 prec**				**1,E-13**		**22,99**	**74634**	**Q15582**	**12 (12 0 0 0 0)**
R.EGVYTVFAPTNEAFR.A	1700,8	-0,3	2	3,E-04	3,19	0,53			
R.FSM*LVAAIQSAGLTETLNR.E	2038,1	-0,5	2	1,E-12	6,10	0,60			
R.GDELADSALEIFK.Q	1407,7	-0,5	2	2,E-07	4,26	0,47			
R.LTLLAPLNSVFK.D	1315,8	-1,1	2	2,E-07	2,98	0,16			
K.SLQGDKLEVSLK.N	1316,7	-0,5	2	9,E-05	3,52	0,36			
K.SPYQLVLQHSR.L	1327,7	-0,5	2	4,E-09	3,38	0,37			
K.STVISYECCPGYEK.V	1692,7	-0,6	2	2,E-09	4,23	0,52			
K.TLFELAAESDVSTAIDLFR.Q	2098,1	-0,6	2	1,E-13	6,45	0,63			
R.YGTLFTM*DR.V	1119,5	-0,4	2	4,E-04	2,61	0,41			
K.YHIGDEILVSGGIGALVR.L	1869,0	-0,2	3	1,E-09	5,52	0,38			
K.YLYHGQTLETLGGK.K	1579,8	-0,2	2	2,E-11	4,63	0,55			
K.YLYHGQTLETLGGKK.L	1707,9	0,1	3	8,E-10	4,03	0,38			
**ANTI-INFLAMMATORY/ANTI-OXIDANT PROTEINS**									
**FSTL1_HUMAN (Q12841) Follistatin-related protein 1 precursor (Follistatin-like**				**1,E-12**		**21,57**	**34963**	**Q12841**	**5 (5 0 0 0 0)**
K.CALEDETYADGAETEVDCNR.C	2317,9	0,6	2	5,E-11	4,89	0,48			
R.GLCVDALIELSDENADWK.L	2048,0	0,7	2	3,E-04	2,66	0,53			
K.ICANVFCGAGR.E	1224,6	-0,4	2	7,E-05	3,52	0,35			
R.LDSSEFLK.F	938,5	0,0	2	3,E-04	2,26	0,23			
K.LSFQEFLK.C	1011,6	-0,8	2	8,E-04	3,23	0,34			
**GSTP1_HUMAN (P09211) Glutathione S-transferase P**				**3,E-11**		**23,04**	**23210**	**P09211**	**3 (3 0 0 0 0)**
K.ALPGQLKPFETLLSQNQGGK.T	2126,2	0,3	3	1,E-09	3,34	0,20			
K.FQDGDLTLYQSNTILR.H	1883,9	-0,4	2	9,E-11	4,93	0,54			
-.PPYTVVYFPVR.G	1337,7	-0,2	2	1,E-06	3,76	0,44			
**PTX3_HUMAN (P26022) Pentraxin-related protein PTX3 precursor**				**6,E-13**		**30,98**	**41993**	**P26022**	**8 (8 0 0 0 0)**
R.ALAAVLEELR.Q	1084,6	-0,5	2	2,E-06	3,56	0,40			
R.GNIVGWGVTEIQPHGGAQYVS.-	2169,1	-0,3	2	4,E-11	2,77	0,46			
R.LAESLARPCAPGAPAEAR.L	1836,9	-0,3	3	3,E-08	4,36	0,31			
R.LESFSACIWVK.A	1339,7	-0,6	2	3,E-05	2,73	0,38			
R.LTGFNIWDSVLSNEEIR.E	1993,0	-0,4	2	2,E-12	5,19	0,47			
R.LTSALDELLQATR.D	1430,8	-0,4	2	4,E-07	5,12	0,39			

	R.M*LLQATDDVLR.G	1290,7	-0,5	2	2,E-06	3,23	0,36				
	R.SWLPAGCETAILFPM*R.S	1864,9	-0,5	2	1,E-08	3,74	0,58				
**PRDX6_HUMAN (P30041) Peroxiredoxin-6**					**5,E-09**		**12,09**	**24888**	**P30041**	**2 (2 0 0 0 0)**	
	K.LPFPIIDDR.N	1085,6	-0,4	2	5,E-05	2,68	0,49				
	-.PGGLLLGDVAPNFEANTTVGR.I	2098,1	-0,4	2	5,E-09	5,37	0,58				
**TRXR1_HUMAN (Q16881) Thioredoxin reductase 1, cytoplasmic precursor (EC 1.8.1.9) (TR)**					**6,E-16**		**19,84**	**54672**	**Q16881**	**5 (5 0 0 0 0)**	
	K.LM*HQAALLGQALQDSR.N	1767,9	-0,9	2	1,E-06	2,23	0,34				
	K.SYDYDLIIIGGGSGGLAAAK.E	1941,0	-1,2	2	6,E-16	5,57	0,56				
	K.VM*VLDFVTPTPLGTR.W	1661,9	-0,7	2	4,E-08	4,67	0,57				
	R.VVAQSTNSEEIIEGEYNTVM*LAIGR.D	2739,4	0,9	3	1,E-09	4,89	0,42				
	R.VVGFHVLGPNAGEVTQGFAAALK.C	2282,2	0,6	3	1,E-14	5,11	0,34				
	**METABOLIC ENZYMES**										
**AK1C1_HUMAN (Q04828) Aldo-keto reductase family 1 member C1**					**5,E-11**		**19,38**	**36765**	**Q04828**	**4 (4 0 0 0 0)**	
	R.HIDSAHLYNNEEQVGLAIR.S	2179,1	0,1	3	5,E-11	6,50	0,54				
	K.LNDGHFM*PVLGFGTYAPAEVPK.S	2376,2	-0,1	3	3,E-07	4,23	0,49				
	K.SKDIVLVAYSALGSHR.E	1715,9	-0,1	3	8,E-07	4,59	0,46				
	R.TPALIALR.Y	854,5	-0,4	2	7,E-05	2,34	0,41				
**ENOA_HUMAN (P06733) Alpha-enolase (EC 4.2.1.11)**					**1,E-30**		**35,80**	**47008**	**P06733**	**19 (19 0 0 0 0)**	
	R.AAVPSGASTGIYEALELR.D	1804,9	-0,3	2	1,E-14	4,93	0,62				2
	K.AGYTDKVVIGM*DVAASEFFR.S	2192,1	1,0	3	2,E-10	4,36	0,48				
	K.AGYTDKVVIGMDVAASEFFR.S	2176,1	-0,4	3	1,E-07	4,87	0,47				
	K.DATNVGDEGGFAPNILENK.E	1960,9	-0,4	2	9,E-13	5,17	0,62				1
	K.DATNVGDEGGFAPNILENKEGLELLK.T	2743,4	-0,1	3	1,E-09	3,65	0,12				
	K.FGANAILGVSLAVCK.A	1519,8	-0,4	2	4,E-10	4,37	0,55				2
	K.FTASAGIQVVGDDLTVTNPK.R	2033,1	-0,4	2	4,E-11	5,07	0,39				
	R.GNPTVEVDLFTSK.G	1406,7	-0,3	2	3,E-07	3,71	0,22				
	R.IGAEVYHNLK.N	1143,6	-0,7	2	7,E-06	3,45	0,35				1
	R.IGAEVYHNLKNVIK.E	1597,9	0,9	3	3,E-07	3,59	0,21				1
	K.LAMQEFMILPVGAANFR.E	1908,0	-0,5	2	1,E-09	4,22	0,53				
	K.LAQANGWGVMVSHR.S	1525,8	-0,3	3	8,E-09	3,41	0,34				
	K.LMIEMDGTENK.S	1280,6	-0,6	2	8,E-05	2,88	0,25				
	R.SGETEDTFIADLVVGLCTGQIK.T	2353,2	-0,5	3	8,E-10	5,88	0,57				2
	K.SKFGANAILGVSLAVCK.A	1735,0	-0,3	3	2,E-07	3,96	0,46				2
	K.VVIGMDVAASEFFR.S	1540,8	0,5	2	1,E-09	3,17	0,53				
	K.VVIGM*DVAASEFFR.S	1556,8	-0,7	2	1,E-11	4,00	0,50				
	K.YDLDFKSPDDPSR.Y	1554,7	-0,3	2	8,E-07	3,67	0,53				
	R.YISPDQLADLYK.G	1425,7	-0,4	2	6,E-09	3,78	0,31				1
**G3P_HUMAN (P04406) Glyceraldehyde-3-phosphate dehydrogenase (EC 1.2.1.12)**					**4,E-15**		**31,27**	**35899**	**P04406**	**6 (6 0 0 0 0)**	
	R.GALQNIIPASTGAAK.A	1411,8	-0,4	2	5,E-06	3,20	0,42				
	K.LISWYDNEFGYSNR.V	1763,8	-0,4	2	3,E-08	4,11	0,61				
	K.LVINGNPITIFQER.D	1613,9	-0,3	2	1,E-10	4,16	0,55				
	K.VIHDNFGIVEGLM*TTVHAITATQK.T	2611,4	0,4	3	4,E-15	5,16	0,52				
	R.VPTANVSVVDLTCR.L	1530,8	-0,4	2	2,E-09	4,54	0,57				
	K.WGDAGAEYVVESTGVFTTM*EK.A	2293,0	-0,2	3	1,E-08	4,81	0,60				

**MDHM_HUMAN (P40926) Malate dehydrogenase, mitochondrial precursor (EC 1.1.1.37)**				**1,E-30**		**29,79**	**35509**	**P40926**	**7 (7 0 0 0 0)**	
R.FVFSLVDAM*NGK.E	1343,7	-0,3	2	3,E-07	3,85	0,50				
K.GCDVVVIPAGVPR.K	1338,7	-0,4	2	3,E-06	4,10	0,60				
K.IFGVTTLDIVR.A	1233,7	-0,3	2	2,E-06	4,18	0,41				
R.LTLYDIAHTPGVAADLSHIETK.A	2365,2	0,4	3	6,E-15	5,62	0,51				
K.M*ISDAIPELK.A	1132,6	-0,4	2	1,E-05	3,20	0,39				
K.TIIPLISQCTPK.V	1370,8	-0,5	2	6,E-08	3,01	0,46				
K.VAVLGASGGIGQPLSLLLK.N	1793,1	-0,5	2	2,E-11	6,12	0,58				
**PGK1_HUMAN (P00558) Phosphoglycerate kinase 1 (EC 2.7.2.3)**				**4,E-15**		**51,13**	**44455**	**P00558**	**11 (11 0 0 0 0)**	
K.ALESPERPFLAILGGAK.V	1769,0	-0,2	2	8,E-11	5,00	0,34				
K.ITLPVDFVTADKFDENAK.T	2023,0	-0,4	2	4,E-15	4,51	0,60				
K.LGDVYVNDAFGTAHR.A	1634,8	-0,4	2	3,E-11	4,27	0,50				1
K.SLLGKDVLFLK.D	1232,8	-0,3	2	3,E-06	3,24	0,39				1
K.TGQATVASGIPAGWM*GLDCGPESSKK.Y	2621,2	0,4	3	1,E-04	3,89	0,51				
K.VLNNM*EIGTSLFDEEGAK.I	1982,9	-0,4	2	2,E-10	4,63	0,54				
K.VLNNMEIGTSLFDEEGAK.I	1966,9	-0,4	2	3,E-13	5,37	0,57				
K.VLPGVDALSNI.-	1097,6	-0,3	1	3,E-04	2,04	0,31				
K.VSHVSTGGGASLELLEGK.V	1740,9	-0,5	2	7,E-15	4,83	0,69				1
K.WNTEDKVSHVSTGGGASLELLEGK.V	2514,2	0,6	3	6,E-10	5,13	0,58				1
K.YSLEPVAVELK.S	1247,7	-0,4	2	5,E-05	3,37	0,37				
**PGAM1_HUMAN (P18669) Phosphoglycerate mutase 1 (EC 5.4.2.1)**				**3,E-11**		**26,22**	**28655**	**P18669**	**5 (5 0 0 0 0)**	
R.ALPFWNEEIVPQIK.E	1683,9	-0,4	2	9,E-08	2,91	0,38				2
R.DAGYEFDICFTSVQK.R	1779,8	-0,5	2	3,E-11	5,20	0,59				
R.FSGWYDADLSPAGHEEAK.R	1979,9	-0,3	3	7,E-11	4,20	0,57				
R.RSYDVPPPPM*EPDHPFYSNISK.D	2589,2	0,5	3	1,E-06	3,62	0,35				1
R.YADLTEDQLPSCESLKDTIAR.A	2425,2	-0,1	3	5,E-11	4,23	0,48				
**KPYM_HUMAN (P14618) Pyruvate kinase isozymes M1/M2 (EC 2.7.1.40) (Pyruvate kinase musc**				**4,E-15**		**29,81**	**57769**	**P14618**	**14 (14 0 0 0 0)**	
R.FDEILEASDGIM*VAR.G	1681,8	-0,8	2	4,E-10	5,72	0,60				
K.GADFLVTEVENGGSLGSK.K	1779,9	-0,9	2	5,E-11	5,90	0,59				
R.GDLGIEIPAEK.V	1141,6	-0,7	2	2,E-05	2,84	0,31				1
K.GVNLPGAAVDLPAVSEK.D	1636,9	-0,8	2	7,E-13	4,93	0,65				
K.ITLDNAYM*EK.C	1213,6	-0,7	2	9,E-05	2,86	0,51				
K.IYVDDGLISLQVK.Q	1462,8	-0,5	2	2,E-05	3,04	0,43				
K.KGVNLPGAAVDLPAVSEK.D	1765,0	0,6	3	8,E-09	4,16	0,34				
R.LAPITSDPTEATAVGAVEASFK.C	2175,1	0,9	3	2,E-06	4,48	0,43				
R.LDIDSPPITAR.N	1197,6	-0,7	2	6,E-06	3,79	0,50				
R.LNFSHGTHEYHAETIK.N	1883,9	0,7	3	1,E-10	4,78	0,58				
R.PVAVALDTK.G	913,5	-0,8	2	1,E-07	3,51	0,49				
R.RFDEILEASDGIM*VAR.G	1837,9	-0,2	3	1,E-07	5,47	0,45				
R.TATESFASDPILYR.P	1570,8	-0,7	2	1,E-07	3,84	0,58				
R.TATESFASDPILYRPVAVALDTK.G	2465,3	0,6	3	9,E-04	3,44	0,30				
**TKT_HUMAN (P29401) Transketolase (EC 2.2.1.1) (TK) [MASS=67877]**				**1,E-15**		**24,10**	**67835**	**P29401**	**9 (9 0 0 0 0)**	
K.ILATPPQEDAPSVDIANIR.M	2020,1	0,6	3	9,E-14	4,33	0,53				
K.ISSDLDGHPVPK.Q	1264,7	-0,8	2	4,E-04	2,88	0,51				

K.LDNLVAILDINR.L	1368,8	-0,7	2	2,E-07	4,63	0,48				
K.NM*AEQIIQEIYSQIQSK.K	2039,0	-1,0	2	2,E-08	4,96	0,38				
K.NSTFSEIFKK.E	1200,6	-0,7	2	5,E-07	2,42	0,25				
K.SKDDQVTVIGAGVTLHEALAAAELLK.K	2649,4	0,9	3	1,E-15	5,41	0,47				
R.SVPTSTVFYPSDGVATEK.A	1884,9	0,4	2	3,E-07	2,45	0,47				
R.TSRPENAIIYNNNEDFQVGQAK.V	2508,2	0,1	3	3,E-12	5,35	0,46				
R.VLDPFTIKPLDR.K	1413,8	1,4	2	5,E-07	3,17	0,36				
**TPIS_HUMAN (P60174) Triosephosphate isomerase (EC 5.3.1.1) (TIM)**				**4,E-13**		**23,60**	**26522**	**P60174**	**3 (3 0 0 0 0)**	
K.QSLGELIGTLNAAK.V	1414,8	0,7	2	4,E-09	4,04	0,60				
K.VTNGAFTGEISPGM*IK.D	1637,8	-0,2	2	2,E-04	3,04	0,61				
K.VVLAYEPVWAIGTGK.T	1602,9	-0,4	2	1,E-08	3,91	0,49				
**CYTOSKELETAL COMPONENTS**										
**ACTB_HUMAN (P60709) Actin, cytoplasmic 1 (Beta-actin)**				**1,E-16**		**26,87**	**41710**	**P60709**	**7 (7 0 0 0 0)**	
R.AVFPSIVGRPR.H	1198,7	-0,2	2	2,E-04	2,73	0,28				5
K.DLYANTVLSGGTTMYPGIADR.M	2215,1	-0,6	2	1,E-06	4,87	0,62				1
K.EITALAPSTMK.I	1161,6	-0,4	2	4,E-04	2,31	0,37				5
R.KDLYANTVLSGGTTM*YPGIADR.M	2359,2	-0,1	3	4,E-07	5,19	0,55				1
K.LCYVALDFEQEM*ATAASSSSLEK.S	2566,2	-0,4	3	1,E-16	5,68	0,39				1
K.SYELPDGQVITIGNER.F	1790,9	-0,4	2	4,E-12	4,26	0,44				5
R.VAPEEHPVLLTEAPLNPK.A	1954,1	-0,3	2	3,E-12	4,26	0,54				1
**ACTN1_HUMAN (P12814) Alpha-actinin-1 (Alpha-actinin cytoskeletal isoform)**				**5,E-14**		**28,03**	**102993**	**P12814**	**4 (4 0 0 0 0)**	
R.FAIQDISVEETSAK.E	1537,8	-0,4	2	2,E-07	3,82	0,51				3
K.IDQLEGDHQLIQEALIFDNK.H	2339,2	0,4	3	5,E-14	5,34	0,60				
K.LASDLLEWIR.R	1215,7	-0,3	2	5,E-06	3,69	0,27				1
K.LM*LLLEVISGER.L	1388,8	0,6	2	5,E-05	2,93	0,25				3
**TBA3_HUMAN (Q71U36) Tubulin alpha-3 chain (Alpha-tubulin 3) (Tubulin B-alpha-1)**				**5,E-10**		**16,72**	**50104**	**Q71U36**	**5 (5 0 0 0 0)**	
R.AVFVDLEPTVIDEVR.T	1701,9	-0,8	2	7,E-06	2,60	0,29				2
K.EIIDLVLDR.I	1085,6	-0,7	2	5,E-04	3,05	0,43				2
R.IHFPLATYAPVISAEK.A	1757,0	-1,0	2	5,E-04	2,44	0,55				4
K.TIGGGDDSFNTFFSETGAGK.H	2007,9	1,1	2	5,E-10	4,06	0,56				3
K.VGINYQPPTVVPGGDLAK.V	1825,0	-0,7	2	2,E-06	3,65	0,49				5
**COTL1_HUMAN (Q14019) Coactosin-like protein**				**3,E-09**		**28,48**	**15804**	**Q14019**	**2 (2 0 0 0 0)**	
R.DDGSAVIWVTFK.Y	1337,7	-0,5	2	3,E-08	4,46	0,54				
K.FALITWIGENVSGLQR.A	1804,0	-0,4	2	3,E-09	5,05	0,51				
**DESP_HUMAN (P15924) Desmoplakin (DP) (250/210 kDa paraneoplastic pemphigus antigen)**				**3,E-09**		**1,36**	**331568**	**P15924**	**3 (3 0 0 0 0)**	
K.IEVLEEELR.L	1129,6	-0,7	2	2,E-05	3,39	0,34				
R.LLEAQIATGGIIDPK.E	1538,9	1,5	2	7,E-07	4,65	0,44				
K.SAIYQLEEEYENLLK.A	1841,9	-0,9	2	3,E-09	5,08	0,58				
**DPYL2_HUMAN (Q16555) Dihydropyrimidinase-related protein 2 (DRP-2) (Collapsin response**				**4,E-12**		**22,38**	**62255**	**Q16555**	**8 (8 0 0 0 0)**	
K.DHGVNSFLVYM*AFK.D	1643,8	-1,0	2	1,E-04	3,86	0,59				
R.DIGAIAQVHAENGDIIAEEQQR.I	2377,2	1,5	3	1,E-09	6,17	0,51				
K.GIQEEM*EALVK.D	1262,6	-0,8	2	3,E-05	3,01	0,40				
R.GSPLVVISQGK.I	1084,6	-0,6	2	2,E-07	3,22	0,44				
R.ILDLGITGPEGHVLSRPEEVEAEAVNR.A	2900,5	0,8	3	4,E-12	3,63	0,46				

K.IVNDDQSFYADIYM*EDGLIK.Q	2365,1	-1,0	2	4,E-10	4,96	0,59				1
K.M*DENQFVAVTSTNAAK.I	1741,8	-0,9	2	8,E-08	4,71	0,55				2
K.VFNLYPR.K	908,5	-0,6	2	7,E-04	2,64	0,39				
**PLAK_HUMAN (P14923) Junction plakoglobin (Desmoplakin-3) (Desmoplakin III)**				**1,E-07**		**5,54**	**81447**	**P14923**	**3 (3 0 0 0 0)**	
K.LLNDEDPVVVTK.A	1341,7	-0,7	2	8,E-07	3,71	0,49				
K.TLVTQNSGVEALIHAILR.A	1935,1	0,9	3	3,E-07	4,48	0,53				
R.VSVELTNSLFK.H	1236,7	0,6	2	1,E-07	3,36	0,39				
**LEG1_HUMAN (P09382) Galectin-1 (Lectin galactoside-binding soluble 1**				**1,E-09**		**11,79**	**14575**	**P09382**	**2 (2 0 0 0 0)**	
R.LNLEAINYM*AADGDFK.I	1800,8	-0,4	2	1,E-08	3,69	0,57				
K.SFVLNLGK.D	877,5	-0,4	2	2,E-05	2,22	0,23				
**MOES_HUMAN (P26038) Moesin**				**2,E-10**		**16,67**	**67647**	**P26038**	**6 (6 0 0 0 0)**	
K.APDFVFYAPR.L	1182,6	-0,2	2	1,E-05	4,25	0,61				2
R.EVWFFGLQYQDTK.G	1660,8	-0,4	2	1,E-08	4,17	0,58				
K.FYPEDVSEELIQDITQR.L	2082,0	-0,3	3	2,E-05	4,36	0,46				
K.IAQDLEM*YGVNYFSIK.N	1906,9	-0,4	2	2,E-10	5,36	0,61				
K.IGFPWSEIR.N	1104,6	-0,4	2	5,E-05	2,36	0,23				2
K.KAPDFVFYAPR.L	1310,7	0,0	2	2,E-06	2,28	0,34				2
**PROF1_HUMAN (P07737) Profilin-1 (Profilin I)**				**3,E-10**		**49,35**	**14914**	**P07737**	**4 (4 0 0 0 0)**	
R.DSLLQDGEFSM*DLR.T	1641,7	-0,4	2	2,E-08	4,73	0,53				
K.DSPSVWAAVPGK.T	1213,6	0,0	2	9,E-05	2,28	0,35				
R.SSFYVNGLTLGGQK.C	1470,8	-0,5	2	5,E-08	4,82	0,46				
K.TFVNITPAEVGVLVGK.D	1643,9	-0,3	2	9,E-07	4,53	0,47				
**RCN1_HUMAN (Q15293) Reticulocalbin-1 precursor**				**9,E-10**	**30,25**	**12,08**	**38866**	**Q15293**	**3 (3 0 0 0 0)**	
K.AADLNGDLTATR.E	1217,6	-0,6	2	1,E-04	2,55	0,33				
K.EIVVLETLEDIDK.N	1515,8	-0,9	2	4,E-08	4,63	0,57				
R.IDNDGDGFVTTEELK.T	1652,8	-1,0	2	5,E-05	2,86	0,29				
**VIME_HUMAN (P08670) Vimentin**				**8,E-14**		**16,06**	**53488**	**P08670**	**5 (5 0 0 0 0)**	
R.EM*EENFAVEAANYQDTIGR.L	2203,0	-0,1	2	8,E-14	4,76	0,68				
K.ILLAELEQLK.G	1169,7	-0,4	2	4,E-06	4,25	0,35				
K.ILLAELEQLKGQGK.S	1539,9	0,3	2	1,E-04	2,60	0,12				
K.LQEEM*LQREEAENTLQSFR.Q	2367,1	0,8	3	2,E-10	4,59	0,63				
K.M*ALDIEIATYR.K	1311,7	-0,4	2	4,E-07	3,97	0,62				1
**VINC_HUMAN (P18206) Vinculin (Metavinculin)**				**1,E-09**		**13,86**	**123591**	**P18206**	**3 (3 0 0 0 0)**	
K.AQQVSQGLDVLTAK.V	1457,8	-1,0	2	1,E-09	4,36	0,57				
K.M*TGLVDEAIDTK.S	1308,6	-0,5	2	1,E-04	3,70	0,50				
R.SLGEISALTSK.L	1105,6	-0,9	2	9,E-05	2,87	0,43				
**WDR1_HUMAN (O75083) WD repeat protein 1 (Actin-interacting protein 1) (AIP1) (NORI-1)**				**2,E-11**		**5,29**	**66021**	**O75083**	**2 (2 0 0 0 0)**	
K.YAPSGFYIASGDVSGK.L	1618,8	-0,8	2	2,E-11	4,19	0,57				
R.FATASADGQIYIYDGK.T	1719,8	-1,0	2	1,E-09	3,32	0,55				
**HEAT SHOCK/PROTEIN FOLDING PROTEINS**										
**GRP78_HUMAN (P11021) 78 kDa glucose-regulated protein precursor**				**7,E-14**		**29,82**	**72289**	**P11021**	**11 (11 0 0 0 0)**	
R.AKFEELNM*DLFR.S	1528,7	0,4	2	4,E-06	3,06	0,25				
R.IEIESFYEGEDFSETLTR.A	2165,0	-0,4	2	7,E-14	6,19	0,65				
R.IINEPTAAAIAYGLDK.R	1659,9	-0,4	2	1,E-12	5,15	0,57				3

R.ITPSYVAFTPEGER.L	1566,8	-0,2	2	1,E-06	3,52	0,43				
K.KKELEEIVQPIISK.L	1654,0	-0,2	3	9,E-04	3,49	0,29				
K.LYGSAGPPPTGEEDTAEKDEL.-	2176,0	-0,4	2	7,E-13	4,37	0,58				
R.NELESYAYSLK.N	1316,6	-0,4	2	6,E-06	3,50	0,35				
K.NQLTSNPENTVFDAK.R	1677,8	-0,3	2	1,E-08	4,43	0,56				
K.SQIFSTASDNQPTVTIK.V	1836,9	-0,6	2	3,E-08	3,96	0,52				
K.TFAPEEISAM*VLTK.M	1552,8	-0,3	2	7,E-08	4,01	0,30				
K.VTHAVVTVPAYFNDAQR.Q	1888,0	0,3	3	8,E-08	4,51	0,42				
**CALU_HUMAN (O43852) Calumenin precursor (Crocalbin)**				**9,E-15**		**27,56**	**37084**	**O43852**	**5 (5 0 0 0 0)**	
K.DIVVQETM*EDIDK.N	1550,7	-0,9	2	9,E-09	4,20	0,41				
K.EEIVDKYDLFVGSQATDFGEALVR.H	2701,3	0,3	3	8,E-05	3,97	0,43				
K.IDGDKDGFVTVDELKDWIK.F	2193,1	0,2	3	5,E-13	5,21	0,45				
K.TFDQLTPEESK.E	1294,6	-0,7	2	1,E-04	2,58	0,51				
K.VHNDAQSFDYDHDAFLGAEEAK.T	2479,1	0,4	3	3,E-06	3,70	0,51				
**CALR_HUMAN (P27797) Calreticulin precursor (CRP55) (Calregulin)**				**2,E-10**		**7,91**	**48112**	**P27797**	**3 (3 0 0 0 0)**	
K.EQFLDGDGWTSR.W	1410,6	0,0	2	8,E-05	2,29	0,46				
R.FYALSASFEPFSNK.G	1607,8	-0,4	2	3,E-10	5,02	0,60				
K.GQTLVVQFTVK.H	1219,7	-0,5	2	2,E-04	2,87	0,48				
**TCPQ_HUMAN (P50990) T-complex protein 1 subunit theta (TCP-1-theta) (CCT-theta)**				**4,E-09**		**12,71**	**59452**	**P50990**	**5 (5 0 0 0 0)**	
K.AIADTGANVVVTGGK.V	1372,7	-0,9	2	4,E-09	4,21	0,54				
K.DM*LEAGILDTYLGK.Y	1554,8	-0,8	2	4,E-08	4,51	0,52				
K.FAEAFEAIPR.A	1150,6	-0,7	2	5,E-07	4,13	0,50				
K.LATNAAVTVLR.V	1128,7	0,0	2	2,E-04	3,35	0,55				
K.LFVTNDAATILR.E	1333,7	0,2	2	5,E-06	3,32	0,49				
**EF1A1_HUMAN (P68104) Elongation factor 1-alpha 1 (EF-1-alpha-1) (Elongation factor 1 A-1)**				**7,E-07**		**14,89**	**50109**	**P68104**	**6 (6 0 0 0 0)**	
R.EHALLAYTLGVK.Q	1314,7	-0,7	2	1,E-03	3,00	0,42				1
K.IGGIGTVPVGR.V	1025,6	0,4	2	3,E-04	2,72	0,25				1
R.LPLQDVYK.I	975,6	-0,2	2	4,E-05	2,44	0,25				1
K.STTTGHLIYK.C	1120,6	-0,7	2	2,E-05	2,75	0,56				1
R.VETGVLKPGM*VVTFAPVNVTTEVK.S	2531,4	0,4	3	4,E-05	4,43	0,45				
K.YYVTIIDAPGHR.D	1404,7	-0,7	2	7,E-07	3,31	0,40				
**HSP7C_HUMAN (P11142) Heat shock 70 kDa protein 8**				**1,E-13**		**32,04**	**70854**	**P11142**	**11 (11 0 0 0 0)**	
R.ARFEELNADLFR.G	1480,8	-0,2	2	1,E-07	4,38	0,45				1
K.DAGTIAGLNVLR.I	1199,7	-0,5	2	2,E-05	2,75	0,27				
K.GPAVGIDLGTTYSCVGVFQHGK.V	2263,1	0,3	3	1,E-13	4,19	0,48				
R.IINEPTAAAIAYGLDK.R	1659,9	-0,4	2	2,E-10	4,79	0,53				3
R.IINEPTAAAIAYGLDKK.G	1788,0	0,2	3	1,E-07	3,59	0,49				1
K.LDKSQIHDIVLVGGSTR.I	1838,0	-0,1	3	3,E-08	4,77	0,49				
K.NQVAM*NPTNTVFDAK.R	1665,8	-0,3	2	1,E-10	4,67	0,62				
K.NSLESYAFNM*K.A	1319,6	-0,4	2	6,E-06	3,65	0,43				
K.SFYPEEVSSM*VLTK.M	1632,8	-0,5	2	1,E-09	3,46	0,51				
R.TTPSYVAFTDTER.L	1487,7	-0,4	2	1,E-08	2,96	0,48				5
K.TVTNAVVTVPAYFNDSQR.Q	1982,0	-0,1	2	2,E-04	3,38	0,39				
**CH60_HUMAN (P10809) 60 kDa heat shock protein, mitochondrial precursor (Hsp60) (60 kDa c**				**3,E-04**		**4,91**	**61016**	**P10809**	**2 (2 0 0 0 0)**	

R.KPLVIIAEDVDGEALSTLVLNR.L	2365,3	-1,1	3	3,E-04	3,38	0,26				
K.VGLQVVAVK.A	912,6	-0,9	2	9,E-04	2,23	0,19				
**HS90A_HUMAN (P07900) Heat shock protein HSP 90-alpha (HSP 86)**				**3,E-12**		**7,73**		**P07900**	**4 (4 0 0 0 0)**	
K.ADLINNLGTIAK.S	1242,7	-0,4	2	5,E-08	3,95	0,47				1
R.ELISNSSDALDKIR.Y	1560,8	-0,3	2	4,E-08	2,74	0,34				
R.NPDDITNEEYGEFYK.S	1833,8	-0,5	2	9,E-12	4,42	0,50				
K.VILHLKEDQTEYLEER.R	2015,0	0,1	3	3,E-12	4,98	0,51				1
**PDIA3_HUMAN (P30101) Protein disulfide-isomerase A3 precursor (EC 5.3.4.1)**				**2,E-11**	**27,67**	**37,82**	**56747**	**P30101**	**10 (10 0 0 0 0)**	
K.DLLIAYYDVDYEK.N	1619,8	0,1	2	2,E-08	4,75	0,47				
R.ELSDFISYLQR.E	1370,7	-0,3	2	1,E-04	3,96	0,46				
R.FLQDYFDGNLK.R	1359,7	-0,1	2	2,E-04	3,28	0,32				
K.FVM*QEEFSR.D	1188,5	-0,8	2	2,E-05	2,80	0,42				
R.GFPTIYFSPANK.K	1341,7	-0,3	2	8,E-04	2,60	0,31				
R.LAPEYEAAATR.L	1191,6	-0,6	2	1,E-05	2,36	0,26				
K.M*DATANDVPSPYEVR.G	1680,8	-0,8	2	2,E-05	3,57	0,55				
K.TFSHELSDFGLESTAGEIPVVAIR.T	2575,3	0,2	3	3,E-10	3,58	0,39				
K.VVVAENFDEIVNNENK.D	1832,9	-0,8	2	2,E-11	5,80	0,46				
K.YGVSGYPTLK.I	1084,6	-0,7	2	2,E-05	3,12	0,40				
**PPIB_HUMAN (P23284) Peptidyl-prolyl cis-trans isomerase B precursor**				**5,E-09**		**9,30**	**22728**	**P23284**	**4 (4 0 0 0 0)**	
K.DFM*IQGGDFTR.G	1302,6	-0,4	2	3,E-05	2,91	0,40				
K.DTNGSQFFITTVK.T	1457,7	-0,4	2	4,E-07	3,73	0,50				
K.TVDNFVALATGEK.G	1364,7	-0,5	2	4,E-06	4,15	0,55				
R.VIFGLFGK.T	880,5	-0,4	2	9,E-05	2,58	0,38				
**PDIA6_HUMAN (Q15084) Protein disulfide-isomerase A6 precursor (EC 5.3.4.1) (Protein disulfi**				**5,E-10**	**15,62**	**29,13**	**48091**	**Q15084**	**5 (5 0 0 0 0)**	
K.DVIELTDDSFDK.N	1396,6	-0,9	2	1,E-07	2,65	0,54				
R.GSTAPVGGGAFPTIVER.E	1615,8	-0,6	2	3,E-05	2,69	0,43				
K.LAAVDATVNQVLASR.Y	1527,8	-0,7	2	5,E-10	4,24	0,55				
R.NSYLEVLLK.L	1078,6	1,3	2	1,E-05	2,75	0,41				
R.TGEAIVDAALSALR.Q	1386,8	-0,7	2	5,E-08	4,05	0,46				
**ENPL_HUMAN (P14625) Endoplasmin precursor (Heat shock protein 90 kDa beta member 1) (9**				**2,E-11**		**17,81**	**92411**	**P14625**	**11 (11 0 0 0 0)**	
R.EEEAIQLDGLNASQIR.E	1785,9	-0,8	2	2,E-11	4,49	0,56				
K.EESDDEAAVEEEEEK.K	1866,7	0,0	2	4,E-06	3,01	0,49				
R.ELISNASDALDK.I	1275,6	0,9	2	2,E-05	3,19	0,49				1
K.FAFQAEVNR.M	1081,5	-0,6	2	2,E-06	2,94	0,36				
K.GVVDSDDLPLNVSR.E	1485,8	-0,8	2	4,E-05	4,00	0,43				
K.LIINSLYK.N	963,6	-0,8	2	1,E-05	2,40	0,35				
R.LISLTDENALSGNEELTVK.I	2046,1	-1,1	2	1,E-08	4,69	0,66				
K.NLLHVTDTGVGM*TR.E	1529,8	0,7	3	5,E-05	3,36	0,50				
R.SGYLLPDTK.A	993,5	1,6	2	8,E-04	2,32	0,27				
K.SILFVPTSAPR.G	1187,7	-0,6	2	2,E-05	3,44	0,46				
K.TETVEEPM*EEEEAAK.E	1737,7	-0,5	2	2,E-06	3,41	0,41				
**OTHER PROTEINS**										
**SYG_HUMAN (P41250) Glycyl-tRNA synthetase (EC 6.1.1.14) (Glycine--tRNA ligase) (GlyRS)**				**4,E-05**		**3,13**	**83087**	**P41250**	**2 (2 0 0 0 0)**	
R.TFFSFPAVVAPFK.C	1457,8	-0,8	2	4,E-05	3,26	0,54				

K.TVNVVQFEPSK.G	1247,7	-0,8	2	1,E-04	3,33	0,37				
**PTD4_HUMAN (Q9NTK5) Putative GTP-binding protein**				**9,E-09**		**8,27**	**44715**	**Q9NTK5**	**3 (3 0 0 0 0)**	
K.IGIVGLPNVGK.S	1066,7	-0,6	2	9,E-05	3,32	0,45				
K.IPAFLNVVDIAGLVK.G	1568,9	-0,3	2	1,E-08	4,19	0,53				
R.NYIVEDGDIIFFK.F	1572,8	-0,5	2	9,E-09	4,57	0,64				
**GDIB_HUMAN (P50395) Rab GDP dissociation inhibitor beta**				**2,E-15**		**15,88**	**50631**	**P50395**	**12 (12 0 0 0 0)**	
K.DLGTESQIFISR.T	1365,7	-0,3	2	9,E-06	4,37	0,46				
K.EIRPALELLEPIEQK.F	1778,0	0,8	3	2,E-06	3,83	0,37				
K.FLVYVANFDEKDPR.T	1712,9	-0,4	2	5,E-08	2,87	0,34				
K.FVSISDLLVPK.D	1217,7	-0,1	2	7,E-04	3,92	0,48				
R.KSDIYVCM*ISFAHNVAAQGK.Y	2255,1	0,5	3	4,E-13	4,81	0,36				
R.LSAIYGGTYM*LNKPIEEIIVQNGK.V	2667,4	0,2	3	2,E-15	5,87	0,58				
R.NPYYGGESASITPLEDLYKR.F	2273,1	0,2	3	1,E-08	4,97	0,54				
K.SPYLYPLYGLGELPQGFAR.L	2141,1	-0,5	2	3,E-08	4,96	0,64				1
R.TDDYLDQPCYETINR.I	1902,8	-0,3	2	1,E-07	4,17	0,51				
R.TYDATTHFETTCDDIK.N	1917,8	0,0	3	9,E-12	3,83	0,48				
K.VPSTEAEALASSLM*GLFEK.R	1996,0	-0,4	2	3,E-12	6,67	0,54				
K.YIAIVSTTVETK.E	1324,7	-0,6	2	2,E-05	2,85	0,48				
**2AAA_HUMAN (P30153) Serine/threonine-protein phosphatase 2A 65 kDa regulatory subunit A**				**5,E-08**		**4,61**	**65051**	**P30153**	**2 (2 0 0 0 0)**	
K.IGPILDNSTLQSEVKPILEK.L	2194,2	0,9	3	5,E-08	4,52	0,39				
K.SALASVIM*GLSPILGK.D	1572,9	-0,2	2	1,E-07	3,41	0,48				

Parameter	Functions
**Reference**	Displays the name of the protein.
**P (pro)**	Displays the probability value for the protein.
**Coverage (%)**	Displays the identifed amino acid as % of the total amino acids.
**MW (Da)**	Molecular weight of the protein (Dalton).
**Accession**	Swiss-Prot primary accession number.
**Peptides**	Displays the number of peptides which allowed the protein identification.
**Hits**	Displays the rank of the identified peptides which allowed the protein identification. (3 0 0 0 0) means that the 3 peptides sequences are identied as first candidates. (0 3 0 0 0) means that the 3 peptides sequences are identified in rank 2 (i.e. as second candidate
Peptide	Displays the peptide sequences.
MH+	Displays the molecular weight (calculated frome the sequence) of the peptides.
DeltaM	Displays the delta between experimental and calculated molecular weight.
z	Displays the charge state of the peptide.
P (pep)	Displays the probability value for the peptide
XC	Displays the cross-correlation value between the observed peptide fragment mass spectrum and the one theoretically predicted*.
ΔCn	Displays the change in cross-correlation between the first and the nth peptide match, where n is the peptide hit number for which ΔCn is calculated.
Count	Displays the number of identical sequences found in the protein database.

*For more information on XC, see An Approach to Correlate Tandem Mass Spectral Data of Peptides with Amino Acid Sequences in a Protein Database; Eng, J.K., McCormick, A.L., and Yates, J.R., III; (1994) J. Am. Soc. Mass Spectrometry 5, 976-989.
