# Supplementary material for: Characterization of human mesenchymal stem cell secretome at early steps of adipocyte and osteoblast differentiation
Source: BMC Mol Biol. 2008 Feb 26;9:26. doi: 10.1186/1471-2199-9-26 (PMC2279142; doi:10.1186/1471-2199-9-26)
Supplement: Additional file 3 — Table 2S: List of primer sequences for gene expression analysis by qRT-PCR and RT-PCR. [file 1471-2199-9-26-S3.PDF]

gene	*forward primer (5'-3')*	*reverse primer (5'-3')*	*T * (°C)*	*size (bp)*	*Accession N°*
**Human**					
b-actin	AGCCATGTACGTTGCTA	AGTCCGCCTAGAAGCA	55	743	NM_001101
UPAR	CTGGAGCTGGTGGAGAAAAG	TGTTGCAGCATTTCAGGAAG	57	406	NM_001005377
TBP	CACGAACCACGGCACTGATT	TTTTCTTGCTGCCAGTCTGGAC	60	88	NM_003194
Alkaline Phosphatase	GCGCAAGAGACACTGAAATATGC	TGGTGGAGCTGACCCTTGAG	60	140	NM_000478
Adiponectin	GCAGTCTGTGGTTCTGATTCCATAC	GCCCTTGAGTCGTGGTTTCC	60	111	NM_004797
hPAI-1	ACCTGGGAATGACCGACATGT	CTCTCGTTCACCTCGATCTTCACT	60	118	NM_000602
**Mouse**					
mTBP	ACCCTTCACCAATGACTCCTATG	ATGATGACTGCAGCAAATCGC	60	189	NM_013684
mPAI-1	CCGATGGGCTCGAGTATGA	TTGTCTGATGAGTTCAGCATCCA	60	136	NM_008871

* Annealing temperature
